# Deep learning for distinguishing normal versus abnormal chest radiographs and generalization to two unseen diseases tuberculosis and COVID-19

**DOI:** 10.1038/s41598-021-93967-2

**Published:** 2021-09-01

**Authors:** Zaid Nabulsi, Andrew Sellergren, Shahar Jamshy, Charles Lau, Edward Santos, Atilla P. Kiraly, Wenxing Ye, Jie Yang, Rory Pilgrim, Sahar Kazemzadeh, Jin Yu, Sreenivasa Raju Kalidindi, Mozziyar Etemadi, Florencia Garcia-Vicente, David Melnick, Greg S. Corrado, Lily Peng, Krish Eswaran, Daniel Tse, Neeral Beladia, Yun Liu, Po-Hsuan Cameron Chen, Shravya Shetty

**Affiliations:** 1 Google Health, Google, Palo Alto, USA; 2 Google Health Via Advanced Clinical, Deerfield, USA; 3 Apollo Radiology International, Hyderabad, India; 4 Northwestern Medicine, Chicago, IL USA

**Keywords:** Medical imaging, Computer science

## Abstract

Chest radiography (CXR) is the most widely-used thoracic clinical imaging modality and is crucial for guiding the management of cardiothoracic conditions. The detection of specific CXR findings has been the main focus of several artificial intelligence (AI) systems. However, the wide range of possible CXR abnormalities makes it impractical to detect every possible condition by building multiple separate systems, each of which detects one or more pre-specified conditions. In this work, we developed and evaluated an AI system to classify CXRs as normal or abnormal. For training and tuning the system, we used a de-identified dataset of 248,445 patients from a multi-city hospital network in India. To assess generalizability, we evaluated our system using 6 international datasets from India, China, and the United States. Of these datasets, 4 focused on diseases that the AI was not trained to detect: 2 datasets with tuberculosis and 2 datasets with coronavirus disease 2019. Our results suggest that the AI system trained using a large dataset containing a diverse array of CXR abnormalities generalizes to new patient populations and unseen diseases. In a simulated workflow where the AI system prioritized abnormal cases, the turnaround time for abnormal cases reduced by 7–28%. These results represent an important step towards evaluating whether AI can be safely used to flag cases in a general setting where previously unseen abnormalities exist. Lastly, to facilitate the continued development of AI models for CXR, we release our collected labels for the publicly available dataset.

## Introduction

Chest radiography (CXR) is a crucial thoracic imaging modality to detect, diagnose, and guide the management of numerous cardiothoracic conditions. Approximately 837 million CXRs are obtained annually worldwide^1^, resulting in a high reviewing burden for radiologists and other healthcare professionals^2,3^. In the United Kingdom, for example, a shortage in the radiology workforce is limiting access to care, increasing wait times, and delaying diagnoses^4^. The need to reduce radiologist workload and improve turnaround time has sparked a surge of interest in developing artificial intelligence (AI)-based tools to interpret CXRs for a broad range of findings^5–7^.

Many algorithms have been developed to detect specific diseases, such as pneumonia, pleural effusion, and fracture, with comparable or higher performance than radiologists^5–10^. However, by virtue of being developed to detect a specific disease, these algorithms may fail to recognize diseases that they were not trained to detect^11–13^. For example, interstitial lung disease may not necessarily trigger a pneumonia detector. Although algorithms of this type may be highly specific, they may not be suitable as comprehensive tools. Moreover, because developing accurate AI algorithms generally requires large labeled datasets, developing algorithms for every potential disease abnormality that may be encountered in a broad clinical setting is impractical. Therefore, a different problem framing is required for use as an effective prioritization tool: algorithms are needed to distinguish normal versus abnormal CXRs more generally, where abnormality is defined as the presence of a clinically actionable finding.

A reliable AI system for distinguishing normal CXRs from abnormal ones can contribute to prompt patient workup and management. There are several use cases for such a system. First, in scenarios with a high reviewing burden for radiologists, the AI algorithm could be used to identify cases that are unlikely to contain findings, empowering healthcare professionals to quickly exclude certain differential diagnoses and allowing the diagnostic workup to proceed in other directions without delay. Cases that are likely to contain findings can be also grouped together for prioritized review, reducing the turnaround time. Second, in settings when clinical demand outstrips availability of radiologists (for example, in the midst of a large disease outbreak), such a system might be used as a frontline point-of-care tool for non-radiologists. Importantly, the AI needs to be evaluated on CXRs with “unseen” abnormalities (i.e. those that it had not encountered during development), to validate its robustness towards new diseases or new manifestations of diseases.

In this work, we developed a deep learning system (DLS) that classifies CXRs as normal or abnormal using data containing a diverse array of CXR abnormalities from 5 clusters of hospitals from 5 cities in India. We then evaluated the DLS for its generalization to unseen data sources and unseen diseases using 6 independent datasets from India, China, and the United States. These datasets comprise two broad clinical datasets, two tuberculosis (TB) datasets, and two coronavirus disease 2019 (COVID-19) datasets with reverse transcription polymerase chain reaction (RT-PCR)-confirmed positive and negative cases. We are also releasing labels we collected (radiologist interpretations) for images in the publicly-available test dataset to facilitate further development and continual research of AI models by the community (see Data availability).

## Results

### Dataset curation

**Figure 1:**
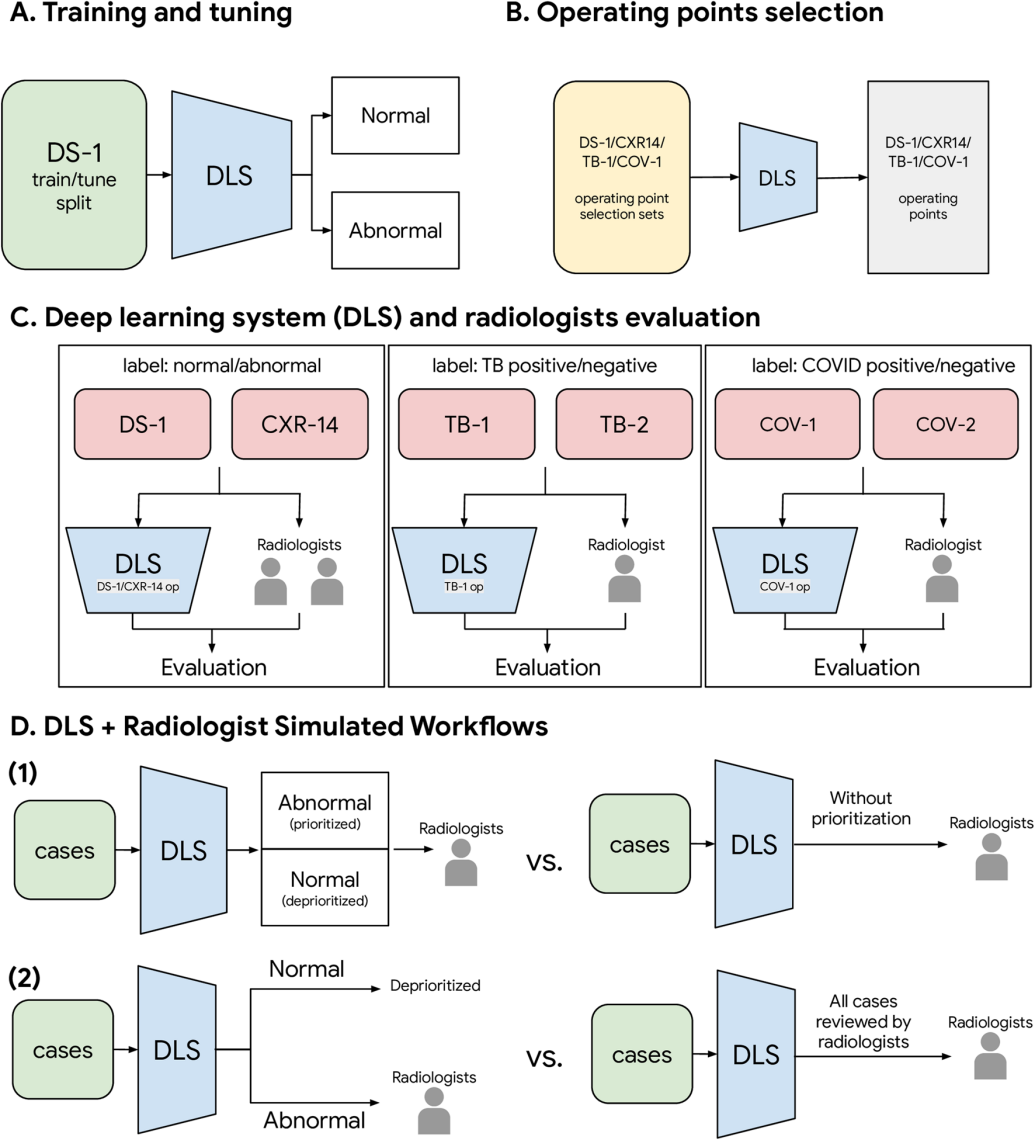
Schematic of the study design, including (**A**) training and tuning, (**B**) operating points selection, (**C**) evaluation on the deep learning system and radiologists, and (**D**) two simulated DLS-assisted workflows. DS-1, CXR-14, TB-1, TB-2, COV-1, COV-2 are abbreviations of the datasets, please see Table 1 and Supplementary Table 1 for details.

Figure 1 shows the overall study design. Our training set consisted of 250,066 CXRs of 213,889 patients from 5 clusters of hospitals from 5 cities in India (Supplementary Table 1, Supplementary Fig. 1). In the training set, all known TB cases were excluded and COVID-19 cases were absent. To evaluate the trained DLS, we used 6 datasets with a total of 11,576 CXRs from 11,298 patients (Table 1, Supplementary Fig. 1). This includes 2 broad clinical datasets (Dataset 1 [DS-1] and ChestX-ray14 [CXR-14], n = 8557 total cases) with 2423 abnormal cases, 2 datasets (TB-1 and TB-2, n = 595 total cases) with 294 TB-positive cases, and 2 datasets (COV-1 and COV-2, n = 2424 total cases) with 873 COVID-19 positive cases. DS-1, COV-1, and COV-2 were obtained from a mixture of general outpatient and inpatient settings and thus represent a wide spectrum of CXRs seen across different populations. Evaluations on these broad datasets mitigates the risk of selecting only the most obvious cases while excluding more difficult images. CXR-14, TB-1, TB-2 were enriched (such as for pneumothoraces in CXR-14; see Supplementary Fig. 2) and were publicly available. Evaluations on these datasets help to validate the DLS’s performance on conditions that would otherwise be rarer, and enables benchmarking with other studies using the same data. To define high-sensitivity and high-specificity operating points for the DLS, we created four small operating point selection datasets for four scenarios: DS-1, CXR-14, TB, and COVID-19; n = 200 cases each (see Fig. 1B and “Operating point selection datasets” section in “Methods”). Across these datasets, we collected 48,877 labels from 31 radiologists for either the reference standard or to serve as a comparison for the DLS (see “Labels” section in “Methods”).

**Table 1:** Data and patient characteristics of the 6 test datasets.

Scenario	Abnormality detection		Unseen disease: TB		Unseen disease: COVID-19	
Dataset	DS-1	CXR-14 (“ChestX-ray14”)	TB-1	TB-2	COV-1	COV-2
Dataset origin	5 clusters of hospitals from 5 cities in India	NIH Clinical Center^7^	A hospital in Shenzhen, China	A hospital in Montgomery, MD, USA	A hospital in Illinois, USA	A hospital in Illinois, USA
No. patients	7747	532	462	133	1819	605
Median age (IQR)	48 (38–58)	49.5 (36–60)	33 (26–43)	40 (28–52)	54 (39–66)	56 (43–68)
No. female (%)	2805 (36.2%)	375 (46.3%)	151 (32.7%)	70 (54.1%)	950 (47.8%)	325 (46.3%)
Race/ethnicity	N/A	N/A	N/A	N/A	White/Caucasian: 769 (42%) Hispanic: 336 (18%) Black/African American: 516 (28%) Asian: 67 (4%) Native Hawaiian/Other Pacific Islander: 3 (0.2%) American Indian/Alaskan Native: 2 (0.1%) Other: 65 (4%) Not available: 61 (3%)	White/Caucasian: 369 (61%) Hispanic: 123 (20%) Black/African American: 58 (10%) Asian: 21 (3%) Native Hawaiian/Other Pacific Islander: 1 (0.2%) American Indian/Alaskan Native: 0 (0%) Other: 24 (4%) Not available: 9 (1%)
No. images	7747	810	462	133	1819	605
PA images	7747	810	462	133	0	0
AP images	0	0	0	0	1819	605
Reference standard	Normal/abnormal based on majority vote of 3 radiologists	Normal/abnormal based on majority vote of 3 radiologists	Radiologists reading without clinical tests	Radiology reports confirmed by clinical tests	COVID-19 status based on RT-PCR test	COVID-19 status based on RT-PCR test
No. abnormal images (%)	1845 (23.8%)	578 (71.4%)	N/A^a^	N/A^a^	N/A^a^	N/A^a^
No. positive images (%, specific disease/finding)	See Supplementary Table 3	See Supplementary Table 4	241 (52.2%, TB)	53 (39.8%, TB)	583 (32.1%, COVID-19)	290 (47.9%, COVID-19)
**Image properties**						
Width (pixels)	512–4400	1143–3827	1130–3001	4020–4892	1024–4200	1024–4200
Height (pixels)	512–4784	966–4715	948–3001	4020–4892	2014–4200	2014–4200
Bit-depth (bits)	12	8	8	8	12	12

N/A indicates information was not available.

^a^Abnormal images in the disease-specific datasets include both those positive for TB or COVID-19, and those with other findings; the numbers of images that contained other findings were not available.

### Classifying CXRs as normal vs abnormal

**Figure 2:**
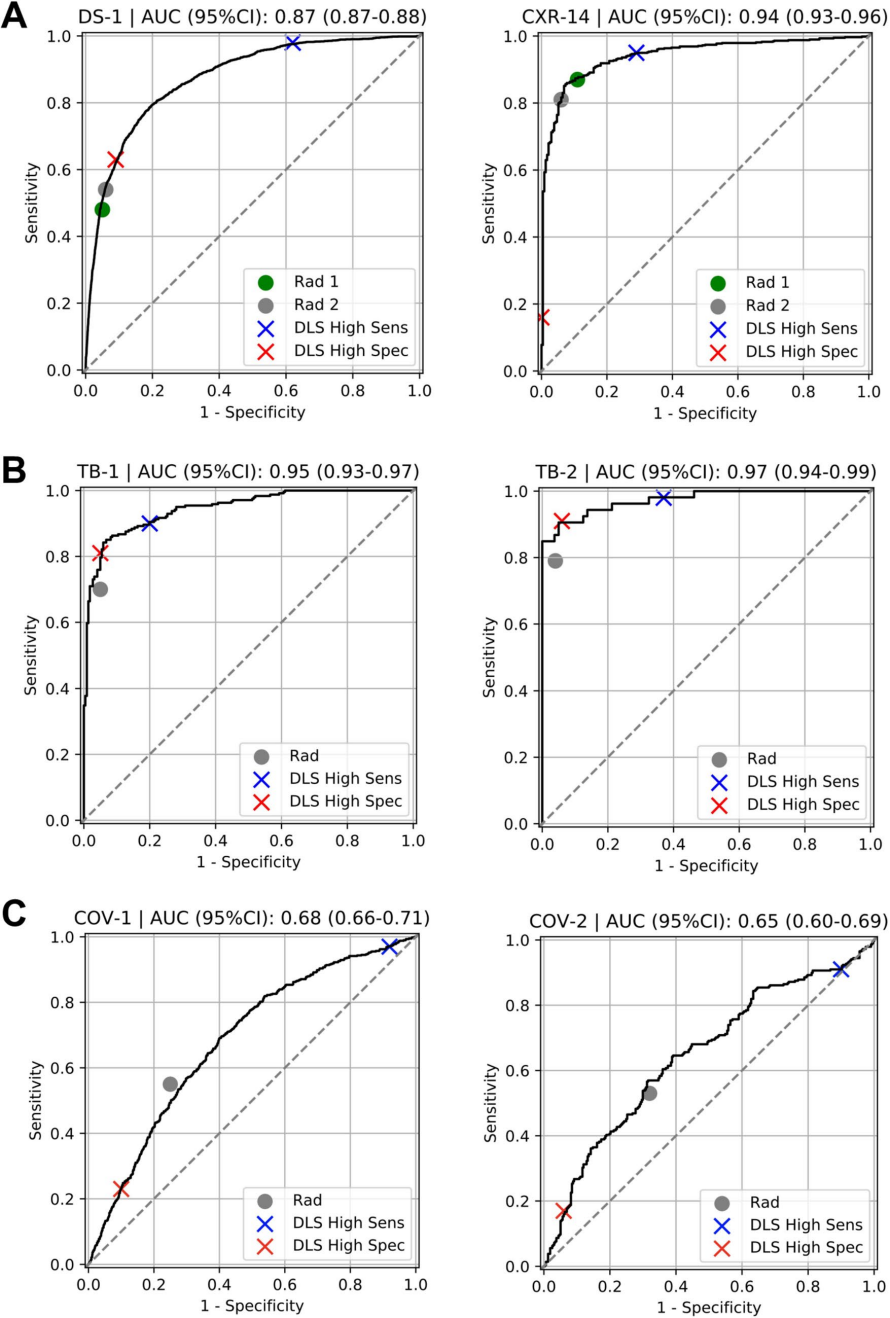
Receiver operating characteristic (ROC) curves for the DLS in distinguishing normal and abnormal CXRs across 6 different datasets. Positive CXRs in DS-1 and CXR-14 contain a mix of multiple labeled abnormalities (Supplementary Table 2). Positive CXRs in the two TB datasets are from patients with tuberculosis. Positive CXRs in the two COVID-19 datasets are from patients with reverse transcription polymerase chain reaction (RT-PCR)-verified COVID-19. Radiologists’ performances in distinguishing the test cases as normal or abnormal are also highlighted in the figures. DLS performance for identifying abnormalities in the TB and COVID-19 datasets (as opposed to the presence or absence of TB or COVID-19) are presented in Supplementary Fig. 6, with AUCs of 0.91-0.93 for TB and 0.86 for COVID-19.

**Figure 3:**
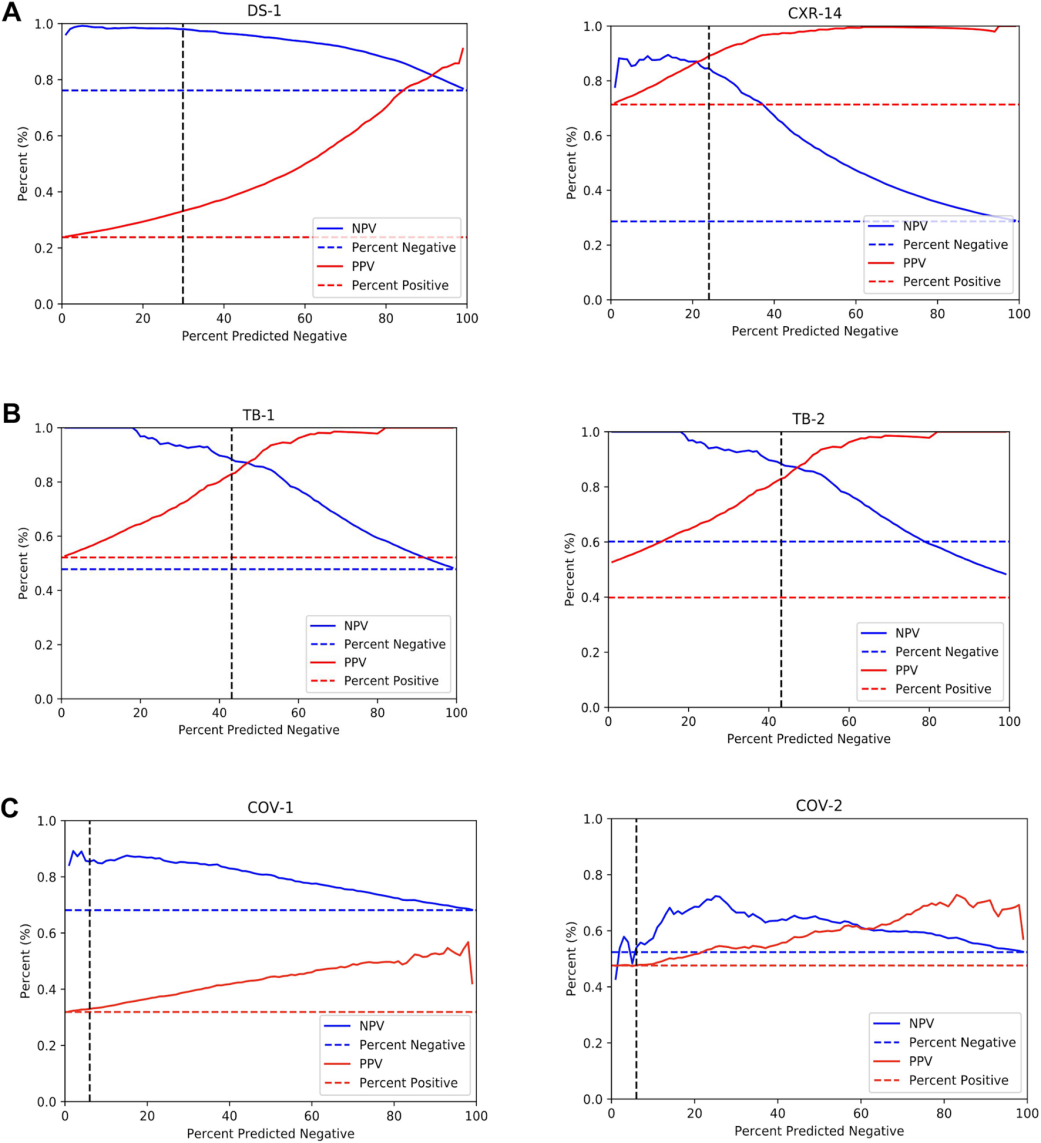
Positive predictive values (PPV) and negative predictive values (NPV) of the DLS across 6 datasets. (**A**) General abnormalities: DS-1 and CXR-14 datasets. (**B**) TB: TB-1 and TB-2. (**C**) COVID-19: COV-1 and COV-2. The horizontal dotted lines represent the prevalence of positive examples (red) and negative examples (blue), which also correspond to random predictions’ PPV and NPV, respectively. The DLS’s NPV converges to the prevalence of negative examples when all examples are predicted as negative, and the DLS’s PPV converges to the prevalence of positive examples when all examples are predicted as positive. The vertical, dotted black lines highlight the selected operating point at 95% sensitivity on the operating point selection sets for each scenario.

The DLS was first evaluated for its ability to classify CXRs as normal or abnormal on the test split of DS-1 and an independent test set CXR-14. We obtained the normal and abnormal labels from the majority vote of three radiologists (see “Labels” section in “Methods”). The percentage of abnormal images were 24% and 71% in DS-1 and CXR-14, respectively (Table 1). The areas under receiver operating characteristic curves (area under ROC, AUC) were 0.87 (95% CI 0.87–0.88) in DS-1 and 0.94 (95% CI 0.93–0.96) in CXR-14 (Table 2, Fig. 2A). To have a comprehensive understanding of the DLS, we measured sensitivity, specificity, negative predictive value (NPV), positive predictive value (PPV), percentage of predicted positives and the percentage of predicted negatives at a high-sensitivity operating point and a high-specificity operating point (“Evaluation metrics” section in “Methods”). With the high-sensitivity operating point (see “Operating point selection” section in “Methods”), the DLS predicted 29.9% of DS-1 and 24.0% of CXR-14 as normal, with NPVs of 0.98 and 0.85, respectively (Table 2). With the high-specificity operating point, the DLS predicted 22.2% of DS-1 and 11.7% of CXR-14 as abnormal, with PPVs of 0.68 and 0.99, respectively (Table 2). The NPVs and PPVs across different operating points are plotted in Fig. 3.

**Table 2:** Quantitative evaluation of DLS in distinguishing normal versus abnormal CXRs across 6 datasets. (A) The DLS’s performance with the high-sensitivity operating point. (B) The DLS’s performance with the high-specificity operating point. The AUC is independent of the operating point and is identical to that in (A).

(A) Scenario	Dataset (reference label used for evaluation)	High-sensitivity operating point (optimizes for NPV)						AUC (95% CI)
		No. predicted negative (%)	NPV (95% CI)	Sensitivity (95% CI)	No. predicted positive (%)	PPV (95% CI)	Specificity (95% CI)	
Abnormality detection	DS-1 (normal/abnormal)	2313 (29.9%)	0.98 (0.97–0.99)	0.98 (0.97–0.98)	5434 (70.1%)	0.33 (0.32–0.34)	0.38 (0.37–0.40)	0.87 (0.87–0.88)
	CXR-14 (normal/abnormal)	194 (24.0%)	0.85 (0.79–0.89)	0.95 (0.93–0.97)	616 (76.0%)	0.89 (0.86–0.91)	0.71 (0.65–0.76)	0.94 (0.93–0.96)
Unseen disease 1: TB	TB-1 (TB status)	199 (43.1%)	0.88 (0.84–0.93)	0.90 (0.87–0.94)	263 (56.9%)	0.83 0.78–0.87 )	0.80 (0.74–0.85)	0.95 (0.93–0.97)
	TB-2 (TB status)	51 (38.3%)	0.98 (0.94–1.0)	0.98 (0.94–1.0)	82 (61.7%)	0.63 (0.51–0.73)	0.63 (0.51–0.73)	0.97 (0.94–0.99)
Unseen disease 2: COVID-19	COV-1 (COVID-19 status)	109 (5.9%)	0.85 (0.78–0.92)	0.97 (0.96–0.98)	1710 (94.0%)	0.33 (0.31–0.35)	0.08 (0.06–0.09)	0.68 (0.66–0.71)
	COV-2 (COVID-19 status)	59 (9.8%)	0.56 (0.43–0.68)	0.91 (0.87–0.94)	546 (90.2%)	0.48 (0.44–0.52)	0.10 (0.07–0.14)	0.65 (0.60–0.69)

(B) Scenario	Dataset (reference label used for evaluation)	High-specificity operating point (optimizes for PPV)						
		No. predicted negative (%)	NPV (95% CI)	Sensitivity (95% CI)	No. predicted positive (%)	PPV (95% CI)	Specificity (95% CI)	
Abnormality detection	DS-1 (normal/abnormal)	6027 (77.8%)	0.89 (0.88–0.90)	0.63 (0.61–0.65)	1720 (22.2%)	0.68 (0.65–0.70)	0.91 (0.90–0.91)	
	CXR-14 (normal/abnormal)	715 (88.3%)	0.32 (0.29–0.36)	0.16 (0.13–0.20)	95 (11.7%)	0.99 (0.96–1.0)	1.0 (0.99–1.0)	
Unseen disease1: TB	TB-1 (TB status)	260 (56.3%)	0.81 (0.76–0.85)	0.81 (0.74–0.84)	202 (43.7%)	0.95 (0.91–0.98)	0.95 (0.92–0.98)	
	TB-2 (TB status)	80 (60.2%)	0.94 (0.88–0.99)	0.91 (0.82–0.98)	53 (39.8%)	0.91 (0.81–0.98)	0.94 (0.88–0.99)	
Unseen disease 2: COVID-19	COV-1 (COVID-19 status)	1558 (85.7%)	0.72 (0.69–0.74)	0.23 (0.20–0.27)	261 (14.3%)	0.52 (0.46–0.58)	0.90 (0.88–0.92)	
	COV-2 (COVID-19 status)	537 (88.8%)	0.55 (0.51–0.59)	0.17 (0.12–0.21)	68 (11.2%)	0.71 (0.59–0.81)	0.94 (0.91–0.96)

To put the performance of the DLS in context, two independent board-certified radiologists reviewed the test splits of both DS-1 and CXR-14. The radiologists had average NPVs of approximately 0.87 and 0.70 and PPVs of 0.75 and 0.96 on DS-1 and CXR-14, respectively (Table 3). The radiologists’ sensitivity and specificity are illustrated on the ROC curves (Fig. 2A).

**Table 3:** Radiologist performance in distinguishing normal and abnormal CXRs across the 6 datasets.

Scenario	Dataset (reference label used for evaluation)	Radiologists					
		No. predicted negative (%)	NPV (95% CI)	Sensitivity (95% CI)	No. predicted positive (%)	PPV (95% CI)	Specificity (95% CI)
Abnormality detection	DS-1 (normal/abnormal)	6567 (84.8%)	0.86 (0.85–0.86)	0.48 (0.46–0.51)	1180 (15.2%)	0.76 (0.74–0.78)	0.95 (0.95–0.96)
		6380 (82.4%)	0.87 (0.86–0.88)	0.54 (0.52–0.57)	1367 (17.6%)	0.74 (0.71–0.76)	0.94 (0.93–0.94)
	CXR-14 (normal/abnormal)	284 (35.1%)	0.73 (0.67–0.77)	0.87 (0.84–0.89)	526 (64.9%)	0.95 (0.93–0.97)	0.89 (0.85–0.93)
		325 (40.1%)	0.67 (0.62–0.72)	0.81 (0.78–0.84)	485 (59.9%)	0.97 (0.96–0.99)	0.94 (0.91–0.97)
Unseen disease: TB	TB-1 (TB status)	282 (61.0%)	0.74 (0.69–0.80)	0.70 (0.65–0.76)	180 (39.0%)	0.93 (0.89–0.97)	0.95 (0.91–0.97)
	TB-2 (TB status)	88 (66.2%)	0.88 (0.81–0.94)	0.79 (0.68–0.90)	45 (33.8%)	0.93 (0.85–1.0)	0.96 (0.92–1.0)
Unseen disease: COVID-19	COV-1 (COVID-19 status)	1194 (65.6%)	0.78 (0.76–0.80)	0.55 (0.51–0.59)	625 (34.4%)	0.51 (0.47–0.54)	0.75 (0.73–0.77)
	COV-2 (COVID-19 status)	352 (58.2%)	0.62 (0.57–0.66)	0.53 (0.48–0.59)	253 (41.8%)	0.60 (0.55–0.66)	0.68 (0.64–0.74)

Radiographic findings vary in their difficulty and importance of detection. Thus we next conducted subgroup analyses for each abnormality listed in Supplementary Table 2. The DLS and radiologists’ performance for distinguishing normal versus abnormal across all individual findings are shown in Supplementary Figs. 2–4 and Supplementary Tables 3 and 4. The DLS showed consistently high NPVs (range 0.93–1.0) with low variability across all findings in both datasets. The radiologists also showed similar NPVs but with higher variability (range 0.86–1.0).

Lastly, for DS-1 and CXR-14, every image was independently reviewed by 3 radiologists to form the reference standard. To understand whether the DLS has learned the intrinsic variability across radiologists, we plotted the distribution of DLS scores stratified by the number of radiologists indicating abnormality in Supplementary Fig. 5. We observed a consistent trend between the DLS scores and the radiologists' discordance across both datasets.

### Performance in the setting of unseen diseases

The DLS was next evaluated on two diseases that it had not been trained to detect (TB and COVID-19) across four disease-specific datasets: TB-1, TB-2, COV-1, and COV-2. In these analyses, the DLS was evaluated against the reference standard for each specific disease (TB or COVID, respectively, see “Labels” section in “Methods”). For TB (where the percentages of disease-positive images were 52% and 40% in TB-1 and TB-2; Table 1), the AUCs were 0.95 (95% CI 0.93–0.97) in TB-1 and 0.97 (95% CI 0.94–0.99) in TB-2 (Table 2, Fig. 2B). At the high-sensitivity operating point, the DLS predicted 43.1% of TB-1 and 38.3% of TB-2 as negative, with NPVs of 0.88 and 0.98, respectively (Table 2A). The NPVs and PPVs across different operating points are also plotted in Fig. 3. However, CXRs that were labeled (TB) negative could nonetheless contain other abnormalities (see “Labels” section in “Methods”). Hence PPVs (Table 2A,B) need to be interpreted with the context that low PPVs for identifying TB-positive radiographs as abnormal do not necessarily reflect the PPV for correctly identifying images with other findings in those datasets (see “Distributional shift between datasets” below). The latter results (DLS performance for identifying abnormalities in TB-1 and TB-2) are presented in Supplementary Fig. 6, with AUCs between 0.91 and 0.93.

Every image in TB1 and TB2 was also annotated as normal or abnormal by one radiologist from a cohort of 8 consultant radiologists from India. The radiologist NPVs were 0.74 and 0.88 and their PPVs were 0.93 and 0.93 on TB-1 and TB-2, respectively (Table 3 and Fig. 2B). Further subgroup analyses comparing the DLS performance with individual radiologists are shown in Supplementary Table 5A,B.

For COVID-19 (where percentage of disease-positive images were 32% and 48% in COV-1 and COV-2; Table 1), the AUCs were 0.68 (95% CI 0.66–0.71) in COV-1 and 0.65 (95% CI 0.60–0.69) in COV-2 (Table 2, Fig. 2A). At the high-sensitivity operating point, the DLS predicts 5.9% of COV-1 and 9.8% of COV-2 as negatives with NPVs of 0.85 and 0.56, respectively (Table 2). The NPVs and PPVs for different operating points are plotted in Fig. 3. Similar to the TB case above, images that were negative for COVID-19 often contained other abnormalities (see “Distributional shift between datasets” section below). The DLS performance for identifying abnormalities in COV-1 and COV-2 are presented in Supplementary Fig. 6, with an AUC of 0.86 in both datasets.

Every image in COV-1 and COV-2 was also reviewed by one radiologist from a cohort of four US board-certified radiologists. The radiologist NPVs were 0.78 and 0.62 and their PPVs were 0.51 and 0.60 on COV-1 and COV-2, respectively (Table 3 and Fig. 2C). Further subgroup analyses comparing the DLS performance with individual radiologists are shown in Supplementary Table 5C,D.

Finally, to better understand the potential impact of the DLS in the setting of imperfect RT-PCR sensitivity, we conducted a subanalysis of COVID-19 cases that had a “false negative” RT-PCR test result on initial testing, defined as a negative RT-PCR test followed by a positive one within five days. In the 21 such cases, the DLS achieved a 95.2% sensitivity, with the CXR taken at the time of the negative test.

### Distributional shifts between datasets

**Figure 4:**
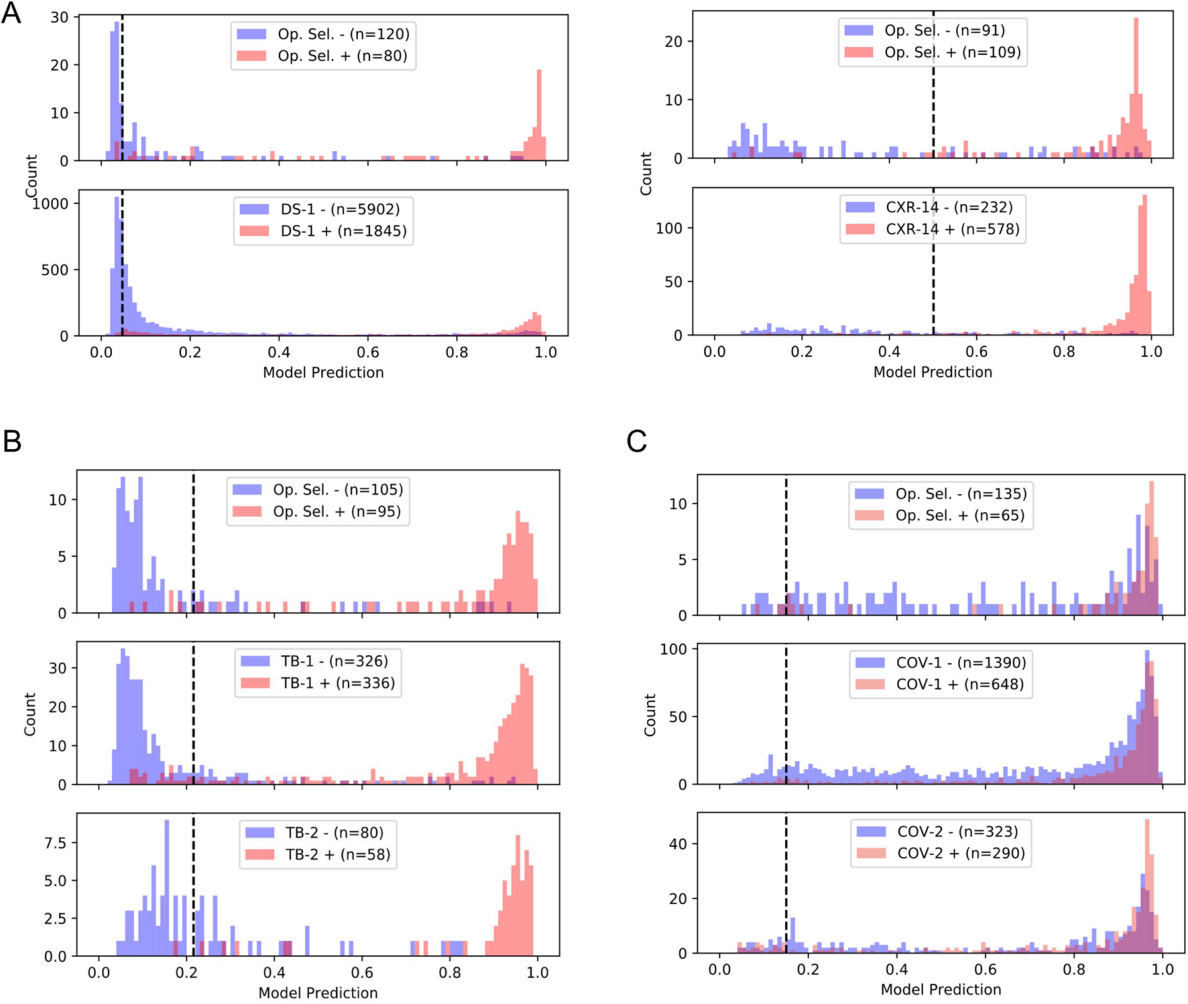
Histogram for the distribution of DLS predicted scores across 6 datasets and their corresponding operating point selection sets: (**A**) DS-1 and CXR-14, (**B**) TB-1 and TB-2, and (**C**) COV-1 and COV-2. Curation of the operating point selection (Op. Sel.) datasets is described in “Operating point selection datasets” in “Methods”. Positive and negative examples are visualized separately in red and blue, respectively. The vertical lines (black) highlight the selected high-sensitivity operating point for each scenario.

To better understand the data shifts between applications (general clinical setting in DS-1 vs. the enriched CXR-14; the broad clinical settings vs. TB; and the broad clinical settings vs. COVID-19), we next examined the distributions of the DLS predictive scores across all 6 test datasets and their corresponding operating point selection sets (Fig. 4, see “Operating point selection datasets” in “Methods”). We observed similarly peaked DLS prediction score distributions (near 1.0) for positive cases—whether for general abnormalities, specific conditions, TB, or COVID-19 (see red histograms in Fig. 4A–C). However, although the distributions for “negative” cases were mostly similar, they did have a small degree of variability, even among datasets of the same scenario from different sites. For example, comparing TB-1 and TB-2 which have similar CXR findings (TB) but were from two independent sites, negative cases in TB-2 had higher scores than in TB-1. Similarly, comparison between COV-1 and COV-2 also shows slight differences in the scores for negative cases. These observations confirm the existence of distributional shifts, suggesting that the scenario-specific operating points are essential, and that even having site-specific operating points may further improve the DLS’s performance.

**Figure 5:**
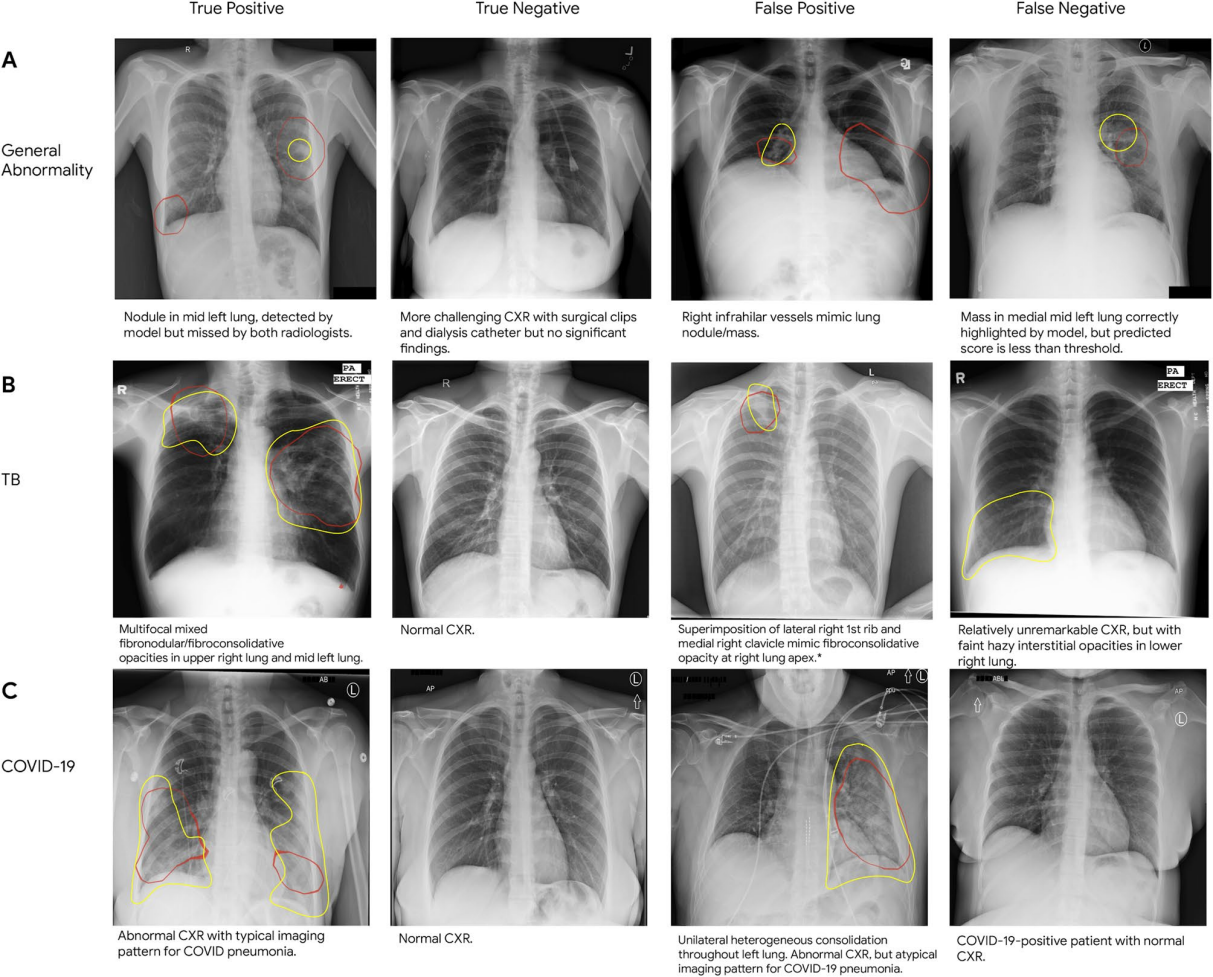
Sample CXRs of true and false positives, and true and false negatives for (**A**) general abnormalities, (**B**) TB, and (**C**) COVID-19. Each image has the class activation map presented as red outlines that indicate the areas the DLS is focusing on for identifying abnormalities, and yellow outlines representing regions of interest indicated by radiologists. Text descriptions for each CXR are below the respective image. Note that the general abnormality false negative example is shown with abnormal class activation maps. However, the DLS predictive score on the case was lower than the selected threshold; hence the image was classified as “normal”. Note that the TB false positive image was saved in the system with inverted colors that were inconsistent with what was specified in the DICOM header tag, and presented to the model that way.

Although scores for positive and the negative cases in DS-1, CXR-14, TB-1, and TB-2 were well-separated, there was significant overlap between the distributions of positive and negative cases for the COVID-19 datasets. In fact, further review of the images revealed that 24.9% of negatives in COV-1 and 31.5% of negatives in COV-2 had other CXR findings, and were thus abnormal. A breakdown of the type of finding in these “negatives” is presented in Supplementary Fig. 7. Examples of challenging cases of each condition and associated saliency maps highlighting the regions with the greatest influence on DLS predictions are presented in Fig. 5.

### Performance of two simulated DLS assisted workflows

**Figure 6:**
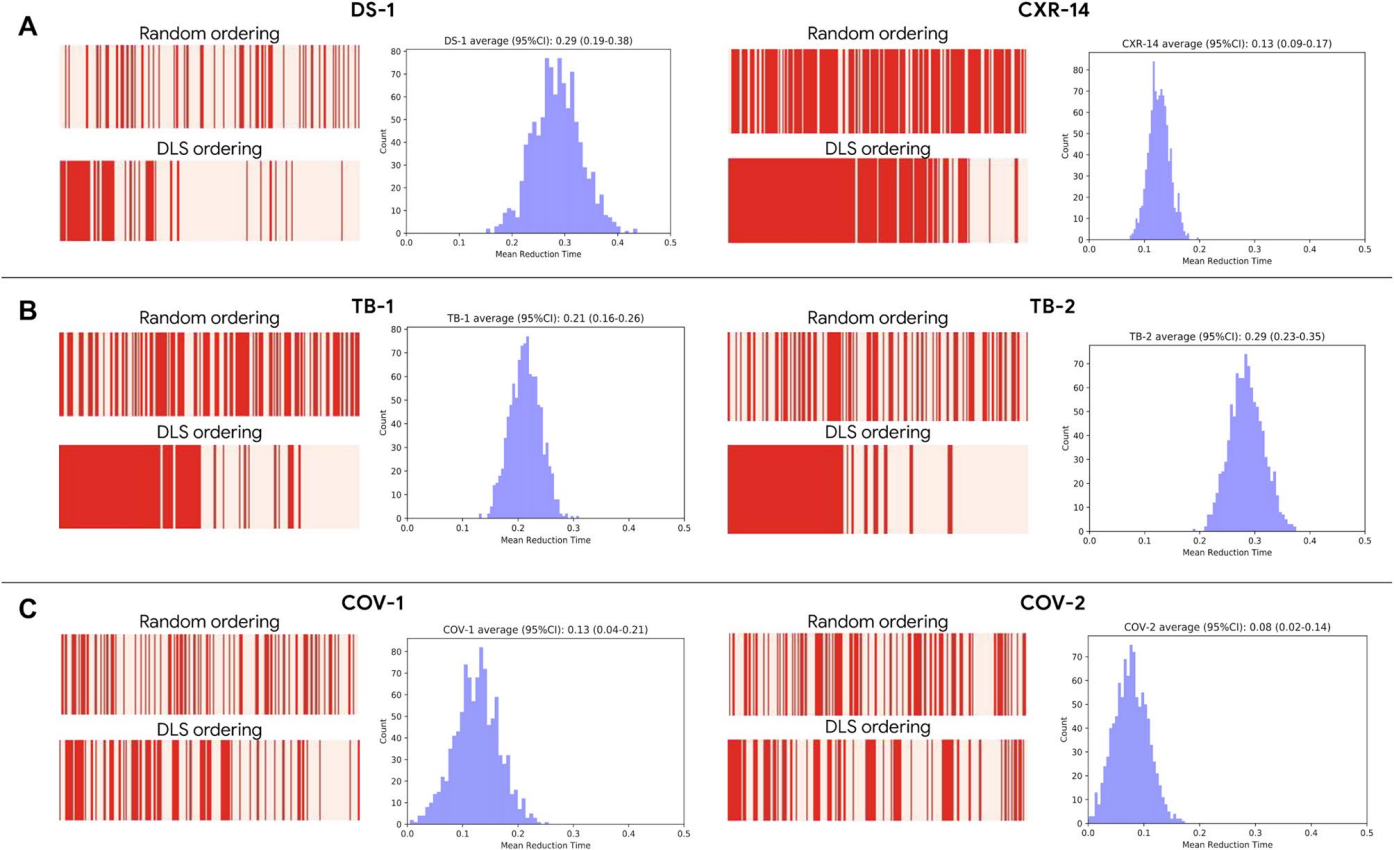
Impact of a simulated DLS-based prioritization in comparison with random review order for (**A**) general abnormalities, (**B**) TB, and (**C**) COVID-19. The red bars indicate sequences of abnormal CXRs in red and normal CXRs in pink; a greater density of red towards the left indicates abnormal CXRs are reviewed sooner than normal ones. The histograms indicate the average improvement in turnaround time.

To understand how the developed DLS can assist practicing radiologists, we investigated two simulated DLS-based workflows. In the first setup, to assist radiologists in prioritizing review of abnormal cases, the DLS sorted cases by the predicted likelihood of being abnormal (Fig. 1D). We measured the differences in expected turnaround time for the abnormal cases with and without DLS prioritization. For simplicity, in this simulation, we assume the same review time for each case, and that the review time per case does not vary based on review order. The DLS-based prioritization reduced the mean turnaround time of abnormal cases by 8–29% for DS-1 and CXR-14, 21–28% for TB-1 and TB-2, and 8–13% for COV-1 and COV-2 (Fig. 6). To understand the effect of relative differences in abnormal vs normal review time, we simulated for a range of different scenarios by varying the time it takes to review an abnormal case with respect to the time it takes to review a normal case (Supplementary Fig. 8). In the second setup, we investigated a simulated sequential reading setup where the DLS identified cases that were unlikely to contain findings, and the radiologist reviewed only the remaining cases (Fig. 1D). Though the deprioritized cases could be reviewed at a later time, we computed the effective immediate performance assuming the DLS-negatives were not yet reviewed by radiologists and considered them to be interpreted as “normal” for evaluation purposes. There were minimal performance differences between radiologists and the sequential DLS-radiologists setup, but the effective “urgent” caseload reduced by 25–30% for DS-1 and CXR-14, about 40% for the TB datasets, and about 5–10% for the COVID-19 datasets (Supplementary Table 6).

## Discussion

We have developed and evaluated a DLS for interpreting CXRs as normal or abnormal, instead of detecting individual abnormalities. We further validated that it generalized with acceptable performance using six datasets: two broad clinical datasets (AUC 0.87 and 0.94), two datasets with one unseen disease (TB; AUC 0.95 and 0.97), and two datasets with a second unseen disease (COVID-19; AUC 0.68 and 0.65).

Generalizability to different datasets and patient populations is critical for evaluation of AI systems in medicine. Studies have shown that many factors might lead to challenges of generalization of AI systems to new populations, such as dataset shift and confounders^14^. Furthermore, with CXRs, as with all medical imagery, the number of potential manifestations is unbounded, especially with the emergence of new diseases over time. Understanding model performance on this set of unseen diseases is an imperative step in developing a robust and clinically useful model that can be trusted in real world situations. In this work, we evaluated the DLS’s performance on 6 independent test sets consisting of different patient populations, spanning three countries, and with two unseen diseases (TB and COVID-19). The DLS’s high sensitivity operating point for ruling out normal CXRs performed on par with board-certified radiologists, with NPVs of 0.85–0.95 (general abnormalities), 0.88–0.98 (TB), and 0.56–0.85 (COVID-19), comparable to radiologist NPVs of 0.67–0.87 (general abnormalities), 0.74–0.88 (TB), and 0.62–0.78 (COVID-19). These results highlight the DLS’s generalizability across real-world dataset shifts, increasing the likelihood of such a system to also generalize to new datasets and new manifestations. The “lower” observed AUCs of the DLS on the COVID-19 datasets were likely caused by our deliberate application of a general abnormality detector to a cohort enriched for patients with a clinical presentation consistent with COVID-19 and thus tested for COVID-19. However, as other acute diseases may share a similar clinical presentation, many cases negative for COVID-19 exhibited abnormal CXR findings that likely triggered the DLS (Fig. 5, Supplementary Fig. 7). Additionally, a substantial number of COVID-19 patients can present with a normal CXR^15^, which would also contribute to a lower observed AUC. Lastly, we expect an improved performance by training the model specifically on a COVID-19 dataset for detecting the disease, and future work is needed to investigate using the current general abnormality model as a pretraining step (i.e. to pre-initialize new networks) for other specific tasks^16^. However, we focused on evaluating a general-abnormal DLS's performance in identifying patients with normal CXRs in a challenging COVID-19 cohort dataset.

In this study, we focused on evaluating the generalizability of the DLS to unseen diseases (TB and COVID) rather than unseen CXR imaging features, in order to assess the clinical relevance of the DLS. Studies have suggested that radiologists’ ability to recognize abnormal imaging features of disease (e.g. consolidation or pleural effusion) on CXR appear relatively independent of experience level, from junior residents through senior faculty^17^. However, proficiency at accurately diagnosing disease on CXR remains strongly tied to experience level^18^. This disparity highlights the value in characterizing an AI system’s ability to detect disease on CXR, in addition to its ability to detect abnormal imaging features.

The variability in patient population and clinical environment across different datasets also meant that the same operating point was unlikely to be appropriate across all settings. For example, a general outpatient setting is substantially less likely to contain CXR findings compared to a cohort of patients with respiratory symptoms or fevers in the midst of the COVID-19 pandemic. Similarly, datasets that are deliberately enriched for specific conditions (CXR-14 and TB) are skewed and are not representative of a general disease screening population. Thus, we used a small number of cases (n = 200) from each setting to determine the operating points specific to that setting. Consistent with this hypothesis, these operating points then generalized well to another dataset, such as from TB-1 to TB-2 and from COV-1 to COV-2. However, further performance improvement is likely possible with site-specific operating point selection sets. We anticipate that this simple operating point selection strategy using a small number of cases may be useful when evaluating an AI system in a new setting, institution, or patient population.

In addition to general performance across the 6 datasets, subgroup analysis of the DLS’ performance on each specific abnormal CXR finding of DS-1 and CXR-14 (Supplementary Tables 3 and 4) revealed consistently high NPVs, suggesting that the DLS was not overtly biased towards any particular abnormal finding. In addition, the DLS outperformed radiologists on atelectasis, pleural effusion, cardiomegaly/enlarged cardiac silhouette, and lung nodules—suggesting that the DLS as a prioritization tool could be particularly valuable in emergency medicine where dyspnea, cardiogenic pulmonary edema, and incidental lung cancer detection are commonly encountered. Furthermore, the DLS also outperformed radiologists in settings where an abnormal chest radiographic finding was present but the abnormality was not one of the predefined chest radiographic findings (e.g. perihilar mass) or radiologists agreed on the presence of a finding but disagreed as to its characterization (indicating case ambiguity; see “Other” in Supplementary Tables 3 and 4). This suggests that the DLS may be robust in the setting of chest radiographic findings that are uncommon or difficult to reach consensus on.

To further evaluate the potential utility of our system, we simulated a setup where the DLS prioritizes cases that are likely to contain findings for radiologists’ review. Our evaluation suggests a potential reduction in turnaround time for abnormal cases by 7–28%, indicating the DLS’s potential to be a powerful first-line prioritization tool. Additionally, we also found that the longer it takes to review an abnormal case, the less reduction in time there was. Whether deployed in a relatively healthy outpatient practice or in the midst of an unusually busy inpatient or outpatient setting, such a system could help prioritize abnormal CXRs for expedited radiologist interpretation. In radiology teams where CXR interpretation responsibilities are shared between general and subspecialist (i.e. cardiothoracic) radiologists, such a system could be used to distribute work. For non-radiologist healthcare professionals, a rapid determination regarding the presence or absence of an abnormality on CXR prevents the release of a patient who needs care and enables alternative diagnostic workup to proceed without delay while the case is pending radiologist review. Finally, a radiologist's productivity might increase by batching negative CXRs for streamlined formal review.

Finally, to facilitate the continued development of AI models for chest radiography, we are releasing our abnormal versus normal labels from 3 radiologists (2430 labels on 810 images) for the publicly-available CXR-14 test set. We believe this will be useful for future work because label quality is of paramount importance for any AI study in healthcare. In CXR-14, the binary abnormal labels were derived through an automated natural language processing (NLP) algorithm on the radiology report^7^. However, editorials have questioned the the quality of labels derived from clinical reports^19^. Hence, in this study we obtained labels from multiple experts to establish the reference standard for evaluation, and a confusion matrix of our majority vote expert labels against the public NLP labels is shown in Supplementary Table 7. We hope that the release of these high-quality labels will aid future work in this area.

Prior studies have demonstrated an algorithm’s potential to differentiate normal and abnormal CXRs^20–25^. Dunnmon et al. showed high diagnostic performance of a developed system in classifying CXRs as normal or abnormal. Hwang et al. evaluated a commercially available system with comparison to radiology residents^22^. Annarumma et al. further demonstrated the system’s utility in a simulated prioritization workflow with three different priority level on a held-out data from the same institution as the training dataset^21^. Our study complements prior works by performing extensive evaluations on model generalizability, including generalization to multiple datasets in different continents, different patient populations settings, and with the presence of unseen diseases. In addition, we also obtained radiologist reviews as benchmarks to understand the DLS’s performance. Lastly, we presented two simulated workflows; one demonstrated reduced turnaround time for abnormal cases, and the other showed comparable performance while reducing effective caseload.

Our study has several limitations. First, there are a wide range of abnormalities and diseases that were not represented among the CXRs available for this study. Although it’s infeasible to exhaustively obtain and annotate datasets for every possible finding, further increasing the conditions and diseases, especially the rare findings, considered in this study could help both in the DLS development and evaluation. Second, we only had labeled data regarding disease-positive and disease-negative for TB and COVID-19. The absence of normal and abnormal labels for the TB and COVID-19 datasets led to added complexity in understanding the performance metrics of PPVs and specificities for these scenarios. The reference standard for the publicly available TB-2 was based on radiologists reading without appropriate clinical tests; hence the performance measure is subject to the diagnoses' accuracy. Third, the follow-up data or information of more sophisticated modalities were not available for DS-1 and CXR-14, limiting the quality of the obtained reference standard. Fourth, to provide a comparison with the DLS, which only had CXRs as input, the radiologists reviewed the cases solely based on CXRs without referencing additional clinical or patient data. In a real clinical setting, this information is generally available, and likely influences a radiologist’s decisions. Fifth, TB cases were excluded from the training and tuning sets by removing all cases indicated as TB-positive or with any reference to TB in the radiology report. Microbiologically verifying the entire training set was infeasible. Hence, there was a potential for leakages of TB positive cases not noted on the radiology reports. Lastly, the results were based on retrospective data. Given the absence of historical reporting timing information, the utility of the DLS-assisted workflows were based on simulation with many assumptions, such as identical radiologist diagnosis regardless of the review order. Additionally, the DLS-assisted workflows did not consider the various degrees of urgency for different diseases, which is an important aspect as a prioritization tool. Hence, the true effects will need to be determined through future evaluation in a prospective setting.

In conclusion, we have developed and evaluated a clinically relevant artificial intelligence model for chest radiographic interpretation and evaluated its generalizability across a diverse set of images in 6 distinct datasets. We hope that the performance analyses reported here along with the release of the expert labels for the publicly available CXR-14 (ChestX-ray14) images will serve as a useful resource to facilitate the continued development of clinically useful AI models for CXR interpretation.

## Methods

### Datasets

In this study, we utilized 6 independent datasets for DLS development and evaluation. The DLS was evaluated in two ways: distinguishing normal vs. abnormal cases in a general setting with multiple radiologist-confirmed abnormalities (first 2 datasets), and in the setting of diseases that the DLS was not exposed to during training (TB was excluded from the train set and COVID-19 was not present; last 4 datasets). All data were stored in the Digital Imaging and Communications in Medicine (DICOM) format and de-identified prior to transfer to study investigators. Details regarding these datasets and patient characteristics are summarized in Table 1, Supplementary Table 1, and Supplementary Fig. 1. This study using de-identified retrospective data was reviewed by Advarra IRB (Columbia, MD), which determined that it was exempt from further review under 45 CFR 46.

#### Train and tune datasets

The first dataset (DS-1) was from five clusters of hospitals across five different cities in India (Bangalore, Bhubaneswar, Chennai, Hyderabad, and New Delhi)^5^. DS-1 consisted of images from consecutive inpatient and outpatient encounters between November 2010 and January 2018, and reflected the natural population incidence of the abnormalities in the populations. All TB cases were excluded and COVID-19 cases were not present. In total, DS-1 originally contained 1,052,274 CXRs from 794,501 patients before exclusions (Supplementary Fig. 1A). This dataset was randomly split into training, tuning, and testing sets in a 0.775:0.1:0.125 ratio while ensuring that images from the same patient remained in the same split. The split is consistent with our previous study^5^. The DLS was developed solely using the training and tuning splits of DS-1. Because outpatient management is primarily done using posterior–anterior (PA) CXRs, while inpatient management is primarily done on anterior–posterior (AP) CXRs, we emphasized PA CXRs in the tune split to better represent an outpatient use case. Both PA and AP images are used in the test datasets.

#### Operating point selection datasets

To select operating points for each of the four scenarios (two general abnormalities, TB, COVID-19), 200 images were randomly selected as the operating point selection sets. For general abnormalities, we selected two independent operating points using 200 randomly sampled images from the DS-1 tune set and 200 randomly sampled images from CXR-14’s publicly-specified combined train and tune set^7,26^. For TB, 200 randomly sampled images from TB-1 were used. For COVID-19, 200 randomly sampled images from COV-1 were used. These images were only used to determine an operating point for that scenario, and once used for operating point selection, were excluded from the test set (Supplementary Fig. 1).

#### Test datasets

Two datasets were used to evaluate the DLS’s performance in distinguishing normal and abnormal findings in a general abnormality detection setting. The first dataset contains 7747 randomly selected PA CXRs from the original test split of the DS-1^5^. These sampled images were expertly labelled as normal or abnormal for the purposes of this study. The second dataset contains 2000 randomly selected CXRs from the publicly-specified test set (25,596 CXRs from 2797 patients) of CXR-14 from the National Institute of Health^7,26^. From these 2000 CXRs (also used in prior work^5^), we removed all the patients younger than 18 years of age and all the AP scans (to focus on an outpatient setting, see tune split procedure above), leaving us with 810 images.

To evaluate the DLS performance in unseen diseases, we curated 2 datasets for TB and 2 datasets for COVID-19 (1 CXR per patient, Supplementary Fig. 1B,C). For TB, one dataset (TB-1) of 462 PA CXRs with 241 confirmed TB positive CXRs was used, from a hospital in Shenzhen, China. Another dataset (TB-2) of 133 PA CXRs with 53 confirmed TB positive CXRs was used from a hospital in Montgomery, MD, USA^27–29^. Both TB datasets are publicly available. For COVID-19, we used 9390 CXRs and 5209 CXRs from all patients who visited two separate hospitals in Chicago in March 2020. Two datasets of 1819 and 605 AP CXRs (with 583 and 290 CXRs with RT-PCR-confirmed COVID-19 positive diagnoses) were curated from the two hospitals: COV-1, COV-2.

### Labels

#### Abnormality labels

For development and evaluation of the DLS, we obtained labels to indicate whether abnormalities were present in each CXR. Each image was annotated as either “normal” or “abnormal”, where an “abnormal” scan is defined as a scan containing at least one clinically-significant finding that may warrant further follow-up. For example, degenerative changes and old fractures were not labeled abnormal because no further management is required. The decision to include abnormal but clinically non-actionable findings as “normal” was based on the intended use case of flagging “abnormality” that requires either downstream action or attention by the clinician.

For the train and tune split of DS-1, we obtained the abnormal and normal labels using NLP (regular expressions) on the radiology reports (Supplementary Table 8). For the normal images, radiology report templates were often used, meaning the same report indicating a normal scan was often used for numerous images. We extracted the most commonly used radiology reports, manually confirmed those that indicated normal reports, and obtained all images that used one of these normal template reports. Examples of these radiology reports along with their frequencies are shown in Supplementary Table 8. For the abnormal images, we obtained all images that did not contain keywords indicating the scan is normal in their respective radiology reports.

For the test sets of DS-1 and CXR-14, a group of US board-certified radiologists reviewed the images at their original resolution to provide reference standard labels. For each image in DS-1, three readers were randomly assigned from a cohort of 18 US board-certified radiologists (range of experience 2–24 years in general radiology). For CXR-14, we obtained labels from three US board-certified radiologists (years of experience: 5, 12, and 24). In both cases, the majority vote of the three radiologists was taken to determine the final reference standard label.

For both DS-1 and CXR-14, in addition to the normal versus abnormal label, we also obtained labels for a selected set of findings present in the abnormal images for subgroup analysis (Supplementary Table 2). Note that the lists of findings for DS-1 and CXR-14 differ. For DS-1, we selected a slightly different list of findings to represent conditions that were more clinically reliable, mutually exclusive, and for which the CXR is reasonably sensitive and specific at characterizing (Supplementary Methods and Supplementary Table 2). Similarly to the normal versus abnormal label, the majority vote was taken for each specific finding. For CXR-14, the differences between the majority voted labels and the publically available labels are shown in a confusion matrix in Supplementary Table 7.

#### TB labels

The first TB dataset^27^ (TB-1) was from Montgomery County, Maryland, USA. The TB positive and negative labels were derived from the radiology reports confirmed by clinical tests and patient history from the tuberculosis control program of the Department of Health and Human Services of Montgomery County, Maryland. The second TB dataset^27^ (TB-2) was from Shenzhen, China. Positive and negative labels for this dataset came from the TB screening results of radiologists reading without appropriate clinical tests in the outpatient clinics in Shenzhen No. 3 People’s Hospital, Guangdong Medical College, Shenzhen, China.

#### COVID-19 labels

For the COVID-19 datasets COV-1 and COV-2, patients with RT-PCR tests and CXRs were included (Supplementary Fig. 1). The COVID-19-positive labels were derived from positive RT-PCR tests. In accordance with current Centers for Disease Control and Prevention (CDC) guidelines^30^, COVID-19-negative labels consisted of CXRs from patients with at least two consecutive negative RT-PCR tests with 12 h apart and no positive test. As false negative rates for RT-PCR have been reported to be ≥ 20% in symptomatic COVID-19-positive patients, CXRs from patients with only one negative RT-PCR test were excluded^31^.

### Deep learning system development

#### Neural network training

We trained a convolutional neural network (CNN) with a single output to distinguish between abnormal and normal CXRs. The CNN uses EfficientNet-B7^32^ as its feature extractor, which was pre-trained on ImageNet^33,34^. Early tuning set results (Supplementary Table 9A) suggested that the EfficientNet-B7 performs better than other advanced networks, hence the decision to use such a network. Since the CNN was pre-trained on three-channel RGB natural images, we tiled the single channel CXR image to three channels for technical compatibility. We trained the CNN using the cross-entropy loss and the momentum optimizer^35^ with a constant learning rate of 0.0004 and a momentum value of 0.9. During training, all images were scaled to 600 × 600 pixels with bilinear interpolation and image pixel values were normalized on a per-image basis to be between 0 and 1. Using higher resolution images (1024 × 1024 pixels) led to non-significantly lower results (Supplementary Table 9B), hence we used 600 × 600 pixels due to its lower computational memory usage. Initializing from ImageNet also appeared to improve results (Supplementary Table 9C). The original bit depth for each image was used (Table 1). For regularization, we applied dropout^36^, with a dropout “keep probability” of 0.5. Furthermore, data augmentation techniques were applied to the input images, including horizontal flipping, padding, cropping, and changes in brightness, saturation, hue, and contrast. All hyperparameters were selected based on the empirical performance on the DS-1 tuning set. We developed the network using TensorFlow and used 10 NVIDIA Tesla V100 graphics processing units for training.

#### Operating point selection

Given a CXR, the DLS predicts a continuous score between 0 and 1 representing the likelihood of the CXR being abnormal. For making clinical decisions, operating points are needed to threshold the scores and produce binary normal or abnormal categorizations. In this study, we selected two operating points (see “Operating point selection datasets” section above), a high sensitivity operating point (95% sensitivity) and a high specificity operating point (95% specificity) for each scenario: general abnormalities for a general clinical setting in DS-1, general abnormalities for an enriched dataset in CXR-14, TB, and COVID-19.

### Comparison with radiologists

To compare the DLS with radiologists in classifying CXRs as normal versus abnormal, additional radiologists reviewed all test images without referencing additional clinical or patient data. All images in the DS-1 and CXR-14 test set were independently interpreted by two board-certified radiologists (with 2 and 13 years of experience), who classified each CXR as normal or abnormal. These radiologists were independent from the cohort of radiologists who contributed to the reference standard labels.

Each image in TB-1 and TB-2 was reviewed by a random radiologist from a cohort of 8 consultant radiologists in India. Each image was annotated as abnormal or normal. Each image in COV-1 and COV-2 was reviewed by one of four board-certified radiologists (with 2, 5, 13, and 22 years of experience). Similarly, each image was annotated as abnormal or normal.

### Two simulated DLS assisted workflows

We simulated two setups in which the DLS was leveraged to optimize radiologists’ workflow (Fig. 1D). In the first setup, we randomly sampled 200 CXRs from each of our 6 datasets to simulate a “batch” workload for a radiologist in a busy clinical environment. For these CXRs, we compared the turnaround time for the abnormal CXRs when (1) they were sorted randomly (to simulate a clinical workflow without the DLS) and (2) when the CXRs were sorted in descending order based on the DLS-predicted scores, such that cases with higher scores appeared earlier. This analysis does not require the selection of an operating point. We repeated each simulation 1000 times per dataset to obtain the empirical distribution of turnaround differences.

In the second setup, we analyzed an extreme use case where the DLS identified CXRs that were unlikely to contain findings using a high sensitivity threshold, and the radiologists only reviewed the remaining cases. All cases skipped by radiologists were labeled negative. We compared the sensitivity between this simulated “reduced workload” workflow and a normal workflow in which the radiologists reviewed all cases.

### Evaluation metrics

To evaluate the DLS across different operating points, we calculated the areas under receiver operating characteristic curves (area under ROC, AUC). To evaluate the performance of the DLS in classifying CXRs as normal or abnormal, we measured negative predictive values (NPV), positive predictive values (PPV), sensitivity, specificity, percentage of predicted negatives, and percentage of predicted positives at a high specificity and a high sensitivity operating point chosen for each scenario (see “Operating point selection” in Deep learning system development. For evaluating the DLS for each individual type of finding, we considered a “each abnormality versus normal” setup where negatives consisted of all normal CXRs, and positives consisted of only the CXRs with that particular finding. As such, specificity values were the same across all findings in a given dataset.

We measured the same set of metrics to evaluate the DLS performance with unseen diseases (TB and COVID-19). However, the ground truth here was defined by either the respective TB or COVID-19 tests, and not whether each image contained any abnormal finding. Thus “negative” TB and COVID-19 cases could still contain other abnormalities.

### Statistical analysis

Confidence intervals (CI) for all evaluation metrics were calculated using the non-parametric bootstrap method with n = 1000 permutations at the image level.

To compare the performance of DLS with the radiologists in a DLS-assisted workflow, non-inferiority tests with paired binary data were performed using the Wald test procedure with a 5% margin^37^. To correct for multiple hypothesis testing, we used Bonferroni correction, yielding α = 0.003125 (one-sided test with α = 0.025 divided by 8 comparisons)^38^.

### Class activation mappings

To provide an approximate visual explanation of how the DLS makes predictions on a small subset of our data, we utilized gradient-weighted class activation mapping (Grad-CAM)^39^ to identify the image regions critical to the model’s decision-making process (Fig. 5). Because overlaying activation maps on an image obscures the original image, a common Grad-CAM visualization shows two images: the original image, and the image with the overlaid activation maps. Here, to balance brevity and clarity, we present the activation maps as outlines highlighting the regions of interest. The outlines were obtained by first using linear interpolation to upsample the low-resolution Grad-CAM feature maps to the size of the original X-rays, resulting in smooth intensity gradations. Next, the majority of the color map is set to a transparent color while a narrow band around 60% of the maximal intensity is opaque to visualize an isoline contour. Conceptually, this is equivalent to taking a horizontal cross-section of the activated maps' three-dimensional contour plot, where the x and y axes represent the spatial location, and the z-axis represents the magnitude of activation. We found this useful as an alternative way to present the Grad-CAM results in a single image. The purpose of these visualizations are for explainability: to visualize and understand the locations influencing model predictions for a few specific examples. The visualizations do not necessarily reflect an accurate segmentation of the lung abnormality.

## Supplementary Information

Supplementary Information.

## Data Availability

Many of the datasets used in this study are publicly available. CXR-14 is a public dataset provided by the NIH (https://nihcc.app.box.com/v/ChestXray-NIHCC)^7,26^. The expert labels we obtained will be made available at https://cloud.google.com/healthcare/docs/resources/public-datasets/nih-chest#additional_labels. TB-1 and TB-2 are publicly available^27,28^. Other than these public datasets, DS-1, COV-1, and COV-2 are owned by their respective institutions. For COV-1 and COV-2 data requests, please contact Dr. Mozziyar Etemadi (mozzi@northwestern.edu). For additional requests, please contact D.T., P.-H.C.C., or S.S.

## Code availability

The deep learning framework used here (TensorFlow v1.15) is available at https://www.tensorflow.org/, https://github.com/tensorflow/tensorflow/tree/r1.15. The neural network architecture EfficientNet (r1.15) is available at https://github.com/tensorflow/tpu/tree/r1.15/models/official/efficientnet. The training code uses the official TensorFlow Estimator API (r2.2) available at https://github.com/tensorflow/tensorflow/tree/r2.2/tensorflow/python/estimator. The Python libraries used for computation and plotting of the performance metrics (SciPy v1.2.1, NumPy v1.16.4, and Matplotlib v3.3.4) are available from https://github.com/scipy/scipy/tree/maintenance/1.2.x, https://github.com/numpy/numpy/tree/maintenance/1.16.x, and https://github.com/matplotlib/matplotlib/tree/v3.3.4-doc, respectively.
