# Supplementary material for: Deep learning for distinguishing normal versus abnormal chest radiographs and generalization to two unseen diseases tuberculosis and COVID-19
Source: Sci Rep. 2021 Sep 1;11:15523. doi: 10.1038/s41598-021-93967-2 (PMC8410908; doi:10.1038/s41598-021-93967-2)
Supplement: Supplementary file 1 — Supplementary Information. [file 41598_2021_93967_MOESM1_ESM.docx]

# Supplementary information

## Supplementary Methods

**List of specific findings for DS-1**

We modified the list of findings from CXR-14 to include conditions that were more likely to be clinically actionable, mutually exclusive, and for which CXR is reasonably sensitive and specific for characterizing (Supplementary Table 2). For example, findings in CXR-14 such as “emphysema” (for which CXR lacks both sensitivity and specificity) and “infiltration” (an ambiguous term that overlaps other CXR-14 findings such as “pneumonia” and “atelectasis”) were replaced by more specific terms. On the other hand, clinically relevant and distinct findings commonly encountered on CXR were also introduced (e.g. “hilar enlargement”, “acute fracture”) or augmented (e.g. “abnormal mediastinal mass/widening” rather than “hiatal hernia”). Our choice of findings for the DS-1 dataset also recognized inherent limitations of CXR for reliably distinguishing between some conditions; hence “focal/multifocal lung opacity” was adopted as a single finding, rather than distinct findings for “consolidation”, “atelectasis”, and “fibroconsolidative opacity”.

**Alternative approach to end-to-end training: lung segmentation**

In addition to the end-to-end approach to detect abnormalities, we also investigated a lung segmentation approach cropping model using a Mask RCNN with a ResNet-101-FPN feature extractor and trained the model on both pixel-level segmentation and bounding boxes as outputs. We then used its bounding box outputs to crop the lungs from each CXR, and used these segmented CXRs as inputs to our classification model. The lung segmentation approach produced tuning AUCs of 0.964 (0.959, 0.971) compared to tuning AUCs of 0.972 (0.967, 0.976) for the end-to-end approach, and was not further pursued.

## Supplementary Figures


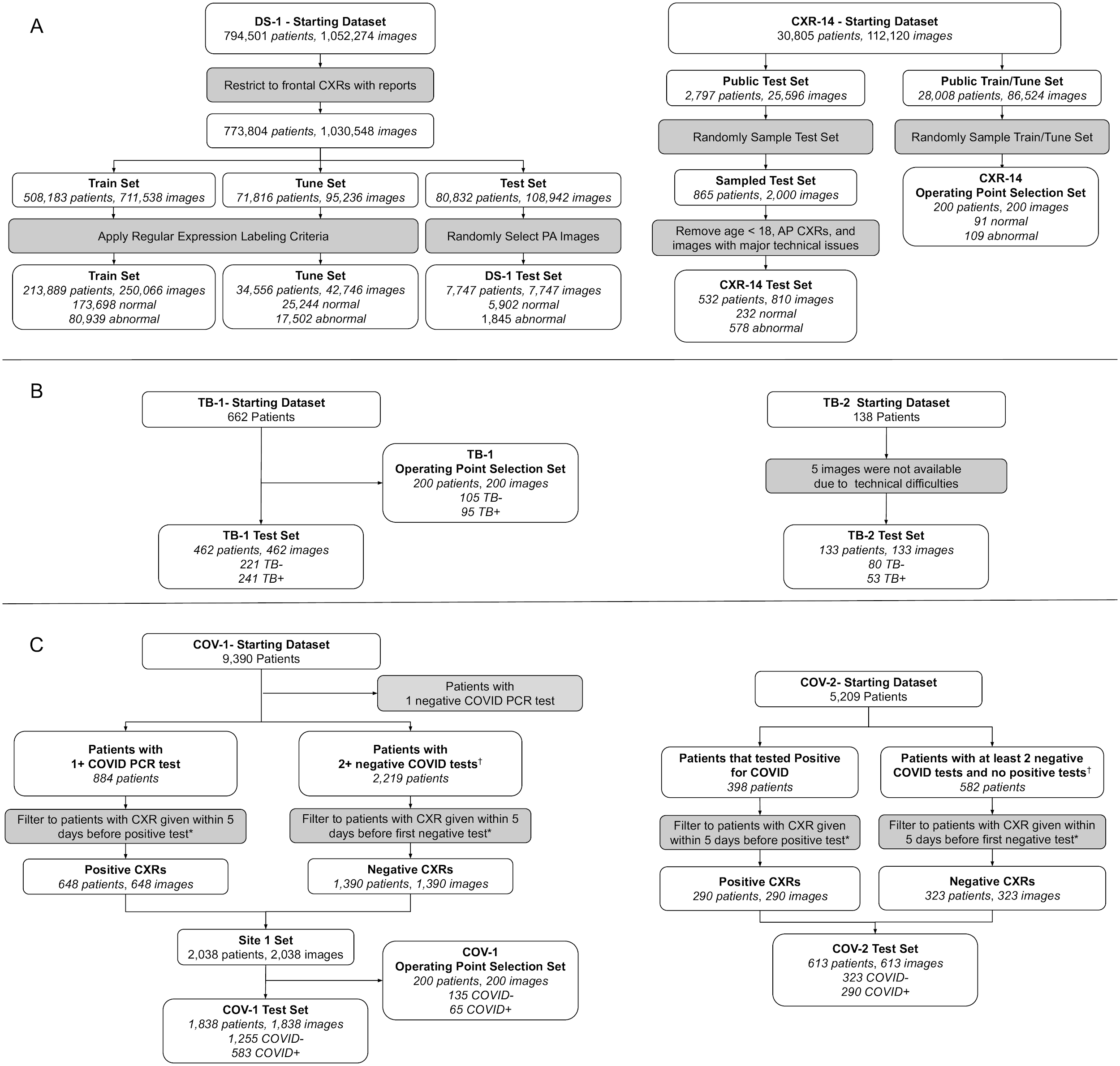


**Sup****plementary Figure 1. The STARD diagrams with inclusion and exclusion criteria for the 6 datasets.** *For COVID-19, the first CXR during the patient’s hospital encounter was selected. ^†^Negative tests had to be administered at least 12 hours apart.


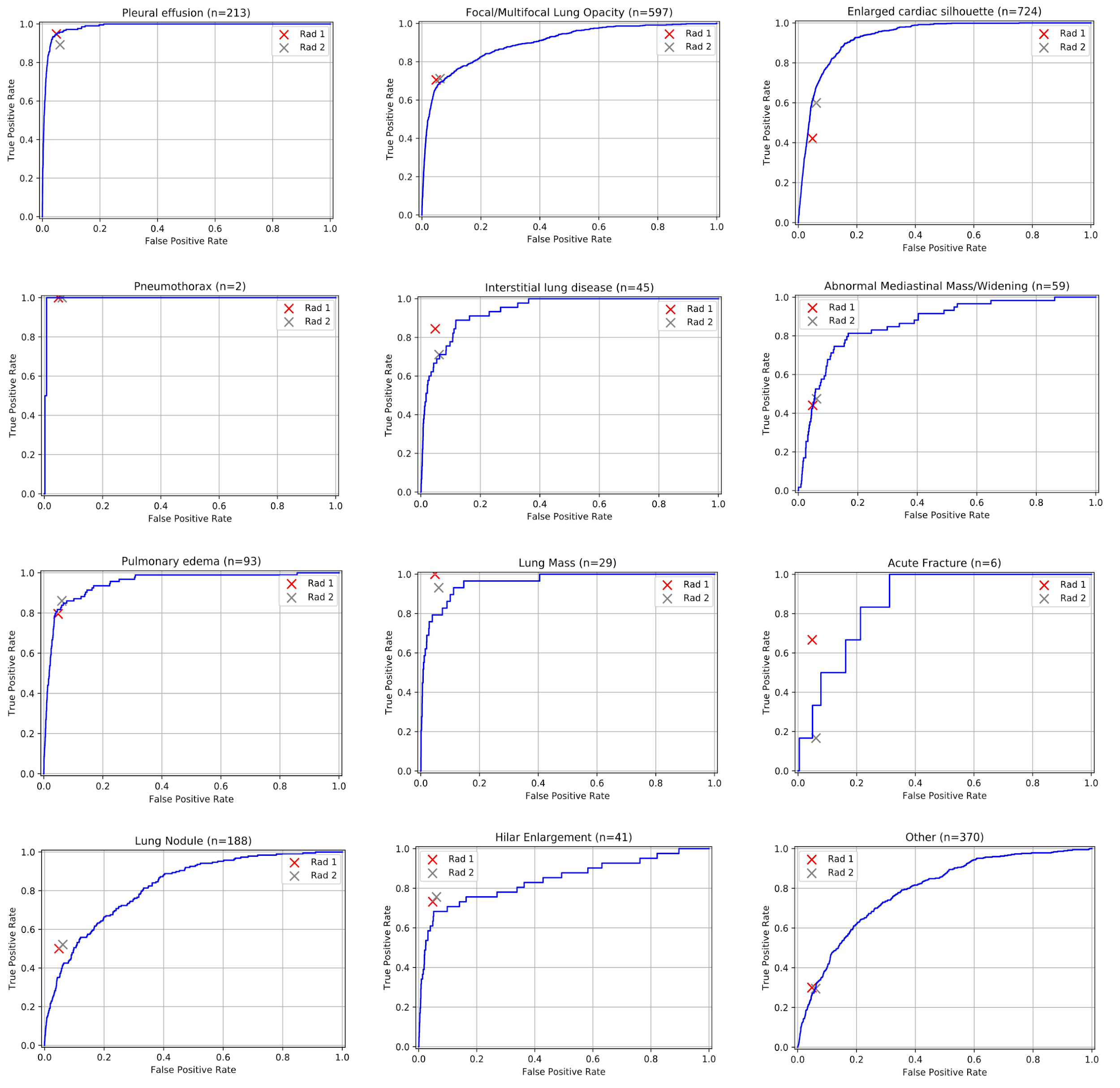


**Supplementary Figure 2. The ROC curves of the DLS in detecting specific findings in DS-1.**


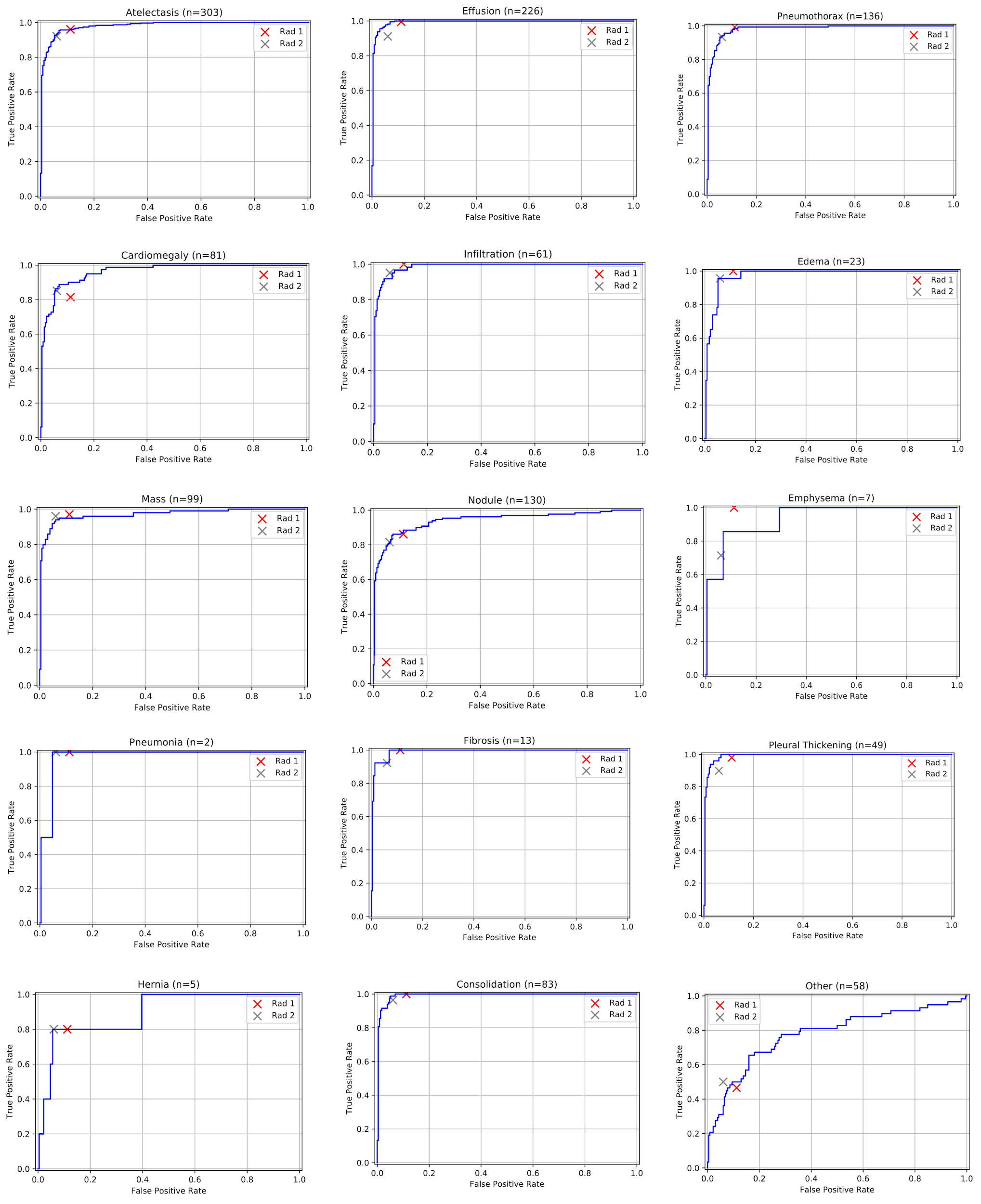


**Supplementary Figure 3. The ROC curves of the DLS in detecting specific findings in CXR-14.**

**
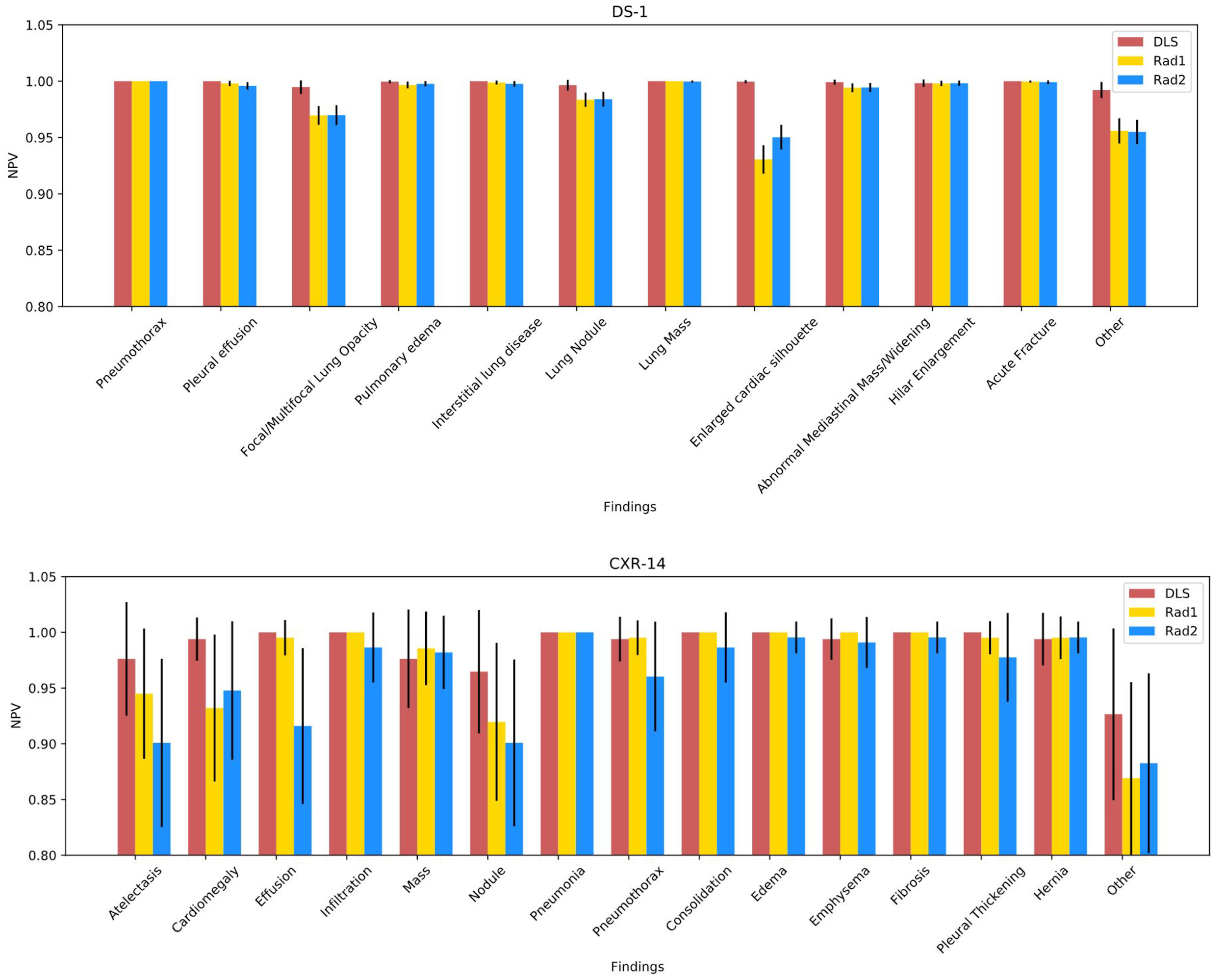
**

**Supplementary Figure 4. Comparison of NPVs between the DLS and the radiologists across specific findings in DS-1 and CXR-14.**

**
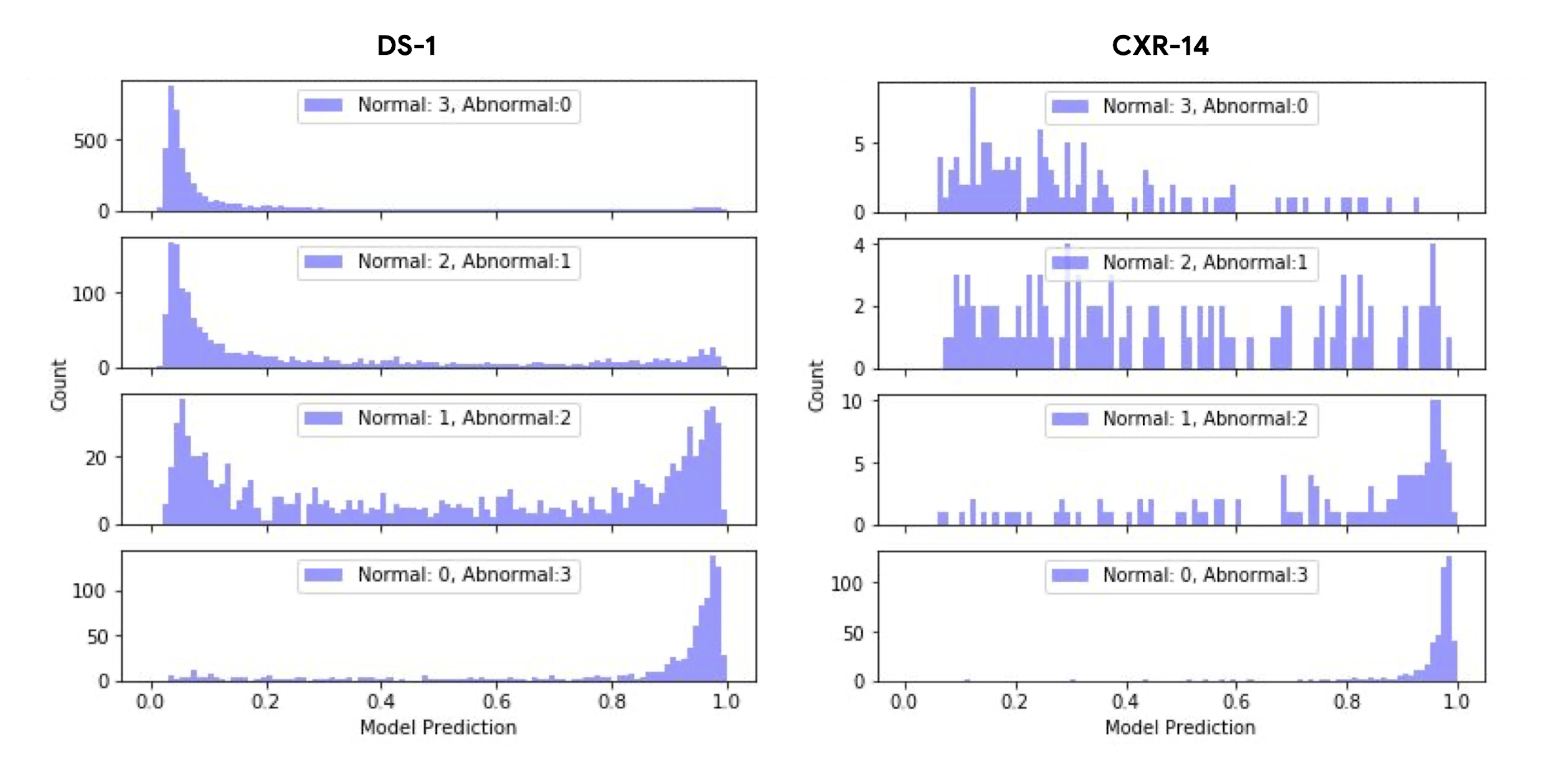
**

**Supplementary Figure 5. Distribution of DLS scores stratified by number of radiologists indicating abnormality.** Every CXR image in DS-1 and CXR-14 were independently reviewed by three radiologists.

##

| 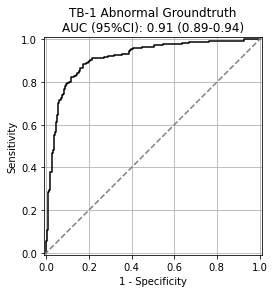 | 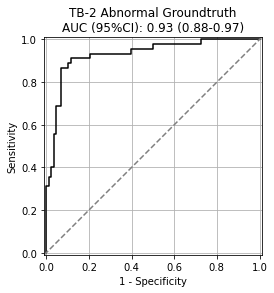 |
| --- | --- |
| 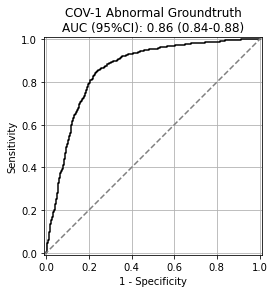 | 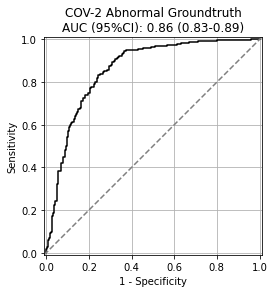 |

## **Supplementary Figure 6. Performance of the DLS classifying CXRs from TB and COVID datasets as normal or abnormal using labels annotated by radiologists as the reference standard.**

**
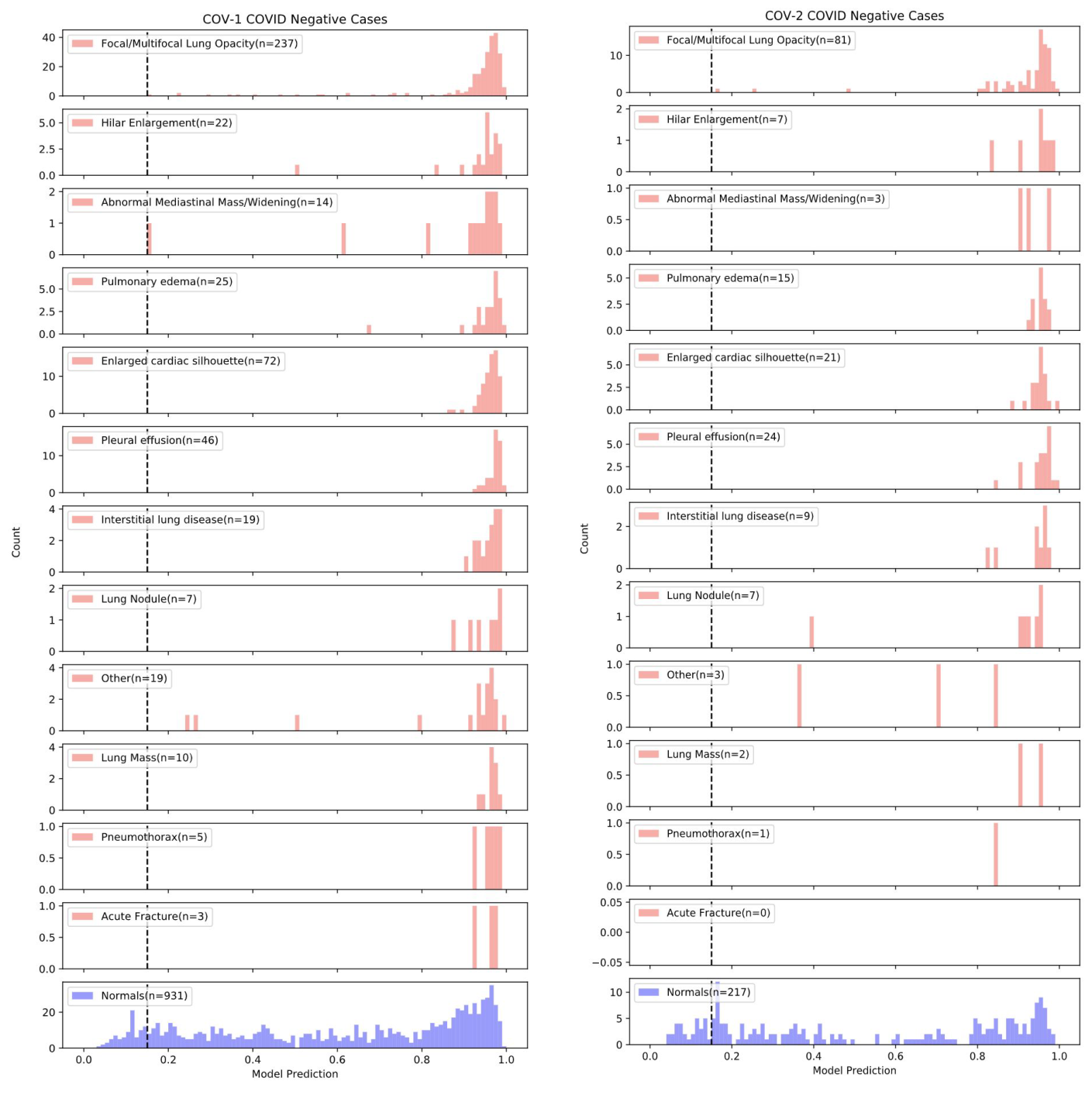
**

**Supplementary Figure 7. Histograms for the distributions of DLS-predicted scores for specific findings in COVID-19-negative cases in COV-1 and COV-2.** The findings were indicated by U.S. board-certified radiologists and were not mutually exclusive as a single case may have had multiple findings.

## **
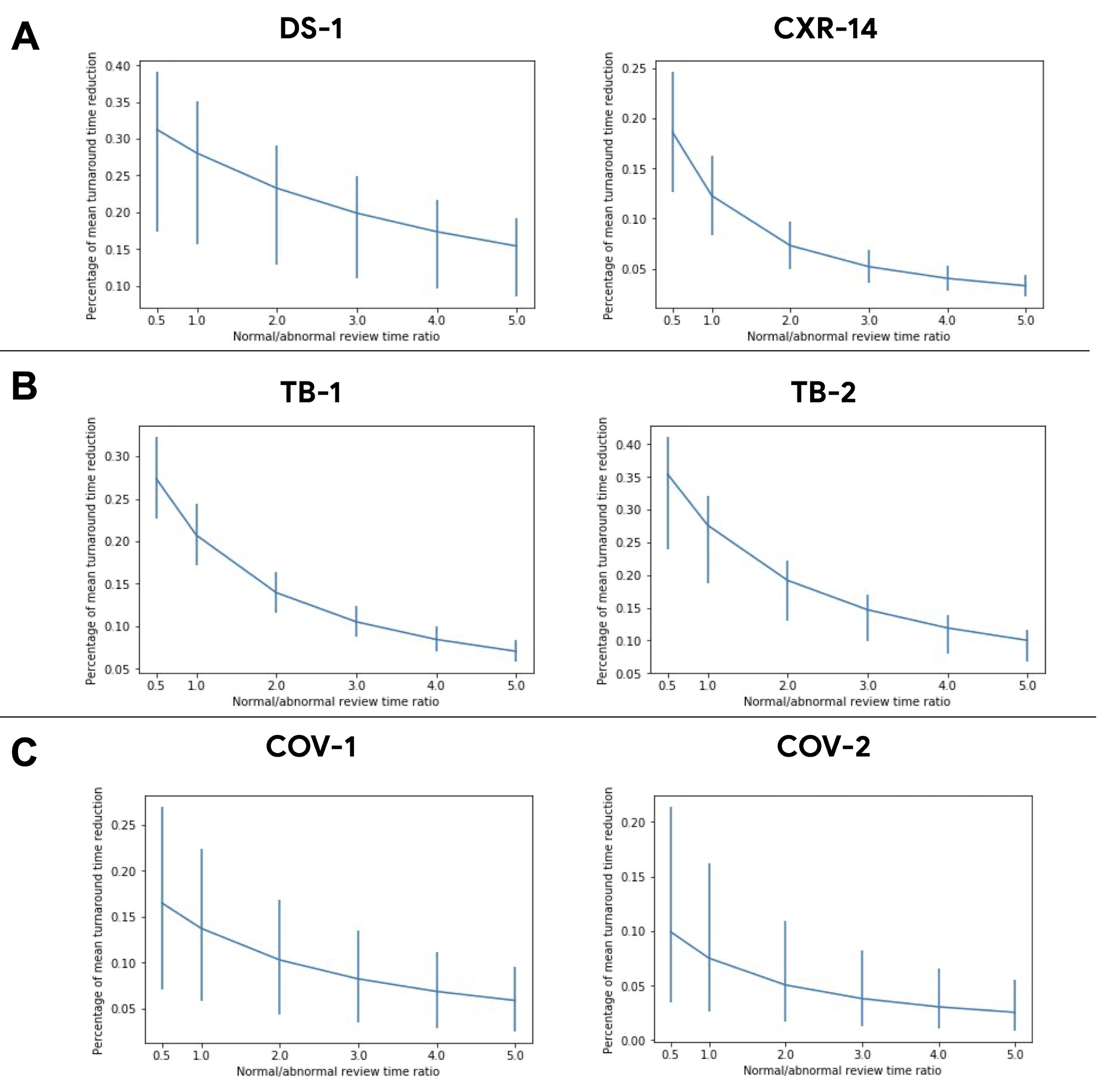
Supplementary Figure 8. Percentage of mean turnaround time across different ratios of reviewing time for abnormal vs normal.**

## Supplementary Tables

**Supplementary Table 1. Data and patient characteristics for the train set, tune set, and operating point selection sets.** *Information not available; for TB and COVID-19 datasets, cases without the disease may still have other abnormal findings.

| Datasets | DS-1  train split | DS-1  tune split | DS-1  operating point selection set | CXR-14 operating point selection set | TB-1  operating point selection set | COV-1  operating point selection set |
| --- | --- | --- | --- | --- | --- | --- |
| Dataset Origin | 5 clusters of hospitals  from 5 cities in India | 5 clusters of hospitals  from 5 cities in India | 5 clusters of hospitals  from 5 cities in India | NIH Clinical Center | a hospital in Shenzhen, China | a hospital in Illinois, USA |
| No. Patients | 213,889 | 34,556 | 200 | 181 | 200 | 200 |
| Median Age (IQR) | 48 (37-58) | 49 (39-59) | 49 (39-59) | 49 (37-59) | 32 (25-42) | 56 (38-67) |
| No. Female (%) | 91,654 (36.7%) | 16092 (37.6%) | 76 (38.0%) | 77 (38.5%) | 62 (31%) | 99 (50.5%) |
| Race / ethnicity | N/A | N/A | N/A | N/A | N/A | White / Caucasian: 89 (45%)  Hispanic: 41 (21%)  Black / African American: 55 (28%)  Asian: 5 (3%)  Native Hawaiian / Other Pacific Islander: 0 (0%)  American Indian / Alaskan Native: 0 (0%)  Other: 8 (4%)  Not Available: 2 (3%) |
| No. Images | 250,066 | 42,746 | 200 | 200 | 200 | 200 |
| PA Images | 202,681 | 42,746 | 200 | 124 | 200 | 0 |
| AP Images | 47,385 | 0 | 0 | 76 | 0 | 200 |
| Number of abnormal images | 80,939 | 17,502 | 80 | 109 | N/A* | N/A* |
| Disease/finding no. of positive images | N/A | N/A | N/A | N/A | TB+:95 | COVID+: 65 |

N/A indicates Information was not available. *For the TB and COVID-19 datasets, cases without the disease may still have other abnormal findings.

**Supplementary Table 2. List of findings in DS-1 and ChestX-ray14.**

| DS-1 Findings | ChestX-ray14 Findings | Comments |
| --- | --- | --- |
| Pneumothorax | Pneumothorax | — |
| Pleural effusion | Effusion | — |
| — | Pleural thickening | CXR is generally nonspecific for pleural thickening, an entity that is often not clinically actionable. |
| — | Emphysema | CXR is not sensitive and not specific for emphysema. |
| — | Infiltration | Deprecated term whose use is discouraged by most subspecialists in thoracic imaging. Definition may vary by individual, and may be taken to imply any/some/all of the following: consolidation, fibrosis, atelectasis, pulmonary edema. |
| Focal/multifocal lung opacity | Consolidation | Consolidation refers to a homogeneous lung opacity that obscures vessel and airway wall margins. Common causes of consolidation include pneumonia, pulmonary edema, and alveolar hemorrhage - entities that are often difficult to distinguish from each other on CXR. As atelectasis and lung fibrosis may often be difficult to distinguish from consolidation on CXR, aggregating these entities may strike a reasonable balance between achieving inter-observer agreement and acknowledging the limitations of CXR. |
|  | Pneumonia |  |
|  | Fibrosis |  |
|  | Atelectasis |  |
| Pulmonary edema | Edema | — |
| Interstitial lung disease | — | Interstitial lung disease is a pathologic entity involving the supporting framework of the lung, and may occur in the setting of occupational inhalation exposures or in older individuals with unexplained chronic dyspnea. |
| Lung nodule | Nodule | — |
| Lung mass | Mass | — |
| Enlarged cardiac silhouette | Cardiomegaly | Enlarged cardiac silhouette can on occasion be caused by pericardial effusion rather than cardiomegaly. As CXR is relatively nonspecific for cardiomegaly vs. pericardial effusion, "enlarged cardiac silhouette" may acknowledge limitations of CXR better. |
| Abnormal mediastinal mass/widening | Hernia | A hiatal hernia is one of many causes of abnormal mediastinal mass/widening, which can range from clinically insignificant to life-threatening. |
|  |  |  |
|  |  |  |
| Hilar enlargement | — | Hilar enlargement is often an actionable imaging finding, which may be caused by malignant, infectious, or inflammatory lymphadenopathy or pulmonary arterial hypertension. |
| Acute fracture | — | Acute fractures are common findings encountered in the emergency/trauma setting and may be associated with other actionable secondary diagnoses (e.g. pneumothorax or hemothorax) or portend more serious underlying injuries. |

**Supplementary Table 3. Performance of DLS on specific findings on DS-1.**

| **Findings** | **# positives** | **High-sensitivity operating point** | | | **High-specificity operating point** | | | **AUC [95% CI]** | **Radiologist 1** | **Radiologist 2** |
| --- | --- | --- | --- | --- | --- | --- | --- | --- | --- | --- |
|  |  | **% predicted negative** | **NPV** | **Sensitivity** | **% predicted positive** | **PPV** | **Specificity** |  |  |  |
| Pneumothorax | 2 | 38.4% | 1 | 1 | 8.7% | 0.004 | 0.91 | 1.0 [0.99, 1.0] | NPV: 1.0  Sens: 1.0  Spec: 0.95  PPV: 0.007 | NPV: 1.0  Sens: 1.0  Spec: 0.94  PPV: 0.005 |
| Pleural Effusion | 213 | 37.1% | 1 | 1 | 11.8% | 0.27 | 0.91 | 0.99 [0.98, 0.99] | NPV: 0.86  Sens: 0.48  Spec: 0.95  PPV: 0.76 | NPV: 1.0  Sens: 0.89  Spec: 0.94  PPV: 0.34 |
| Focal or Multifocal Lung Opacity | 597 | 35.1% | 0.99 | 0.98 | 14.6% | 0.44 | 0.91 | 0.90 [0.89, 0.91] | NPV: 0.97  Sens: 0.71  Spec: 0.95  PPV: 0.60 | NPV: 0.97  Sens: 0.71  Spec: 0.94  PPV: 0.54 |
| Pulmonary Edema | 93 | 37.8% | 1.0 | 0.99 | 9.92% | 0.13 | 0.91 | 0.95 [0.92, 0.97] | NPV: 1.0  Sens: 0.8  Spec: 0.95  PPV: 0.21 | NPV: 1.0  Sens: 0.86  Spec: 0.94  PPV: 0.18 |
| Interstitial Lung Disease | 45 | 38.1% | 1.0 | 1.0 | 9.23% | 0.06 | 0.91 | 0.94 [0.91,0.96] | NPV: 1.0  Sens: 0.84  Spec: 0.95  PPV: 0.12 | NPV: 1.0  Sens: 0.71  Spec: 0.94  PPV: 0.08 |
| Lung Nodule | 188 | 37.3% | 1.0 | 0.96 | 8.84% | 0.14 | 0.91 | 0.82 [0.80, 0.85] | NPV: 0.98  Sens: 0.50  Spec: 0.95  PPV: 0.25 | NPV: 0.98  Sens: 0.52  Spec: 0.94  PPV: 0.21 |
| Lung Mass | 29 | 38.2% | 1.0 | 1.0 | 9.09% | 0.04 | 0.91 | 0.96 [0.93,0.98] | NPV: 1.0  Sens: 1.0  Spec: 0.95  PPV: 0.09 | NPV: 1.0  Sens: 0.93  Spec: 0.94  PPV: 0.07 |
| Enlarged Cardiac Silhouette | 724 | 34.2% | 1.0 | 1.0 | 16.0% | 0.50 | 0.91 | 0.93 [0.92, 0.94] | NPV: 0.93  Sens: 0.42  Spec: 0.95  PPV: 0.52 | NPV: 0.95  Sens: 0.60  Spec: 0.94  PPV: 0.54 |
| Abnormal Mediastinal Mass/Widening | 59 | 38.1% | 1.0 | 0.97 | 9.23% | 0.06 | 0.91 | 0.87 [0.82, 0.91] | NPV: 0.99  Sens: 0.43  Spec: 0.95  PPV: 0.08 | NPV: 0.99  Sens: 0.46  Spec: 0.94  PPV: 0.07 |
| Hilar Enlargement | 41 | 38.1% | 1.0 | 0.90 | 9.14% | 0.05 | 0.91 | 0.84 [0.76, 0.92] | NPV: 1.0  Sens: 0.73  Spec: 0.95  PPV: 0.09 | NPV: 1.0  Sens: 0.76  Spec: 0.94  PPV: 0.08 |
| Acute Fracture | 6 | 38.4% | 1.0 | 1.0 | 8.77% | 0.005 | 0.91 | 0.86 [0.76, 0.96] | NPV: 1.0  Sens: 0.67  Spec: 0.95  PPV: 0.01 | NPV: 1.0  Sens: 0.17  Spec: 0.94  PPV: 0.002 |
| Other (i.e., not a finding listed above) | 377 | 36.4% | 0.99 | 0.95 | 10.4% | 0.20 | 0.91 | 0.79 [0.77, 0.81] | NPV: 0.96  Sens: 0.31  Spec: 0.95  PPV: 0.29 | NPV: 0.95  Sens: 0.29  Spec: 0.94  PPV: 0.23 |

**Supplementary Table 4. Performance of DLS on specific findings on CXR-14.**

| **Findings** | **# positives** | **High-sensitivity operating point** | | | **High-specificity operating point** | | | **AUC [95% CI]** | **Radiologist 1** | **Radiologist 2** |
| --- | --- | --- | --- | --- | --- | --- | --- | --- | --- | --- |
|  |  | **% predicted negative** | **NPV** | **Sensitivity** | **% predicted positive** | **PPV** | **Specificity** |  |  |  |
| Atelectasis | 303 | 31.4% | 0.98 | 0.99 | 14.4% | 0.99 | 1.0 | 0.98 [0.97, 0.99] | NPV: 0.94  Sens: 0.96  Spec: 0.89  PPV: 0.92 | NPV: 0.90  Sens: 0.92  Spec: 0.94  PPV: 0.95 |
| Cardiomegaly | 81 | 52.8% | 0.99 | 0.99 | 5.75% | 0.94 | 1.0 | 0.96 [0.94, 0.98] | NPV: 0.93  Sens: 0.81  Spec: 0.89  PPV: 0.72 | NPV: 0.95  Sens: 0.85  Spec: 0.94  PPV: 0.83 |
| Effusion | 226 | 35.8% | 1.0 | 1.0 | 15.7% | 0.99 | 1.0 | 0.99 [0.97, 1.0] | NPV: 1.0  Sens: 1.0  Spec: 0.89  PPV: 0.90 | NPV: 0.92  Sens: 0.91  Spec: 0.94  PPV: 0.94 |
| Infiltration | 61 | 56.0% | 1.0 | 1.0 | 4.10% | 0.92 | 1.0 | 0.99 [0.97, 1.0] | NPV: 1.0  Sens: 1.0  Spec: 0.89  PPV: 0.70 | NPV: 0.99  Sens: 0.95  Spec: 0.94  PPV: 0.81 |
| Mass | 99 | 50.8% | 0.98 | 0.96 | 6.65% | 0.95 | 1.0 | 0.97 [0.95, 0.99] | NPV: 0.99  Sens: 0.97  Spec: 0.89  PPV: 0.79 | NPV: 0.98  Sens: 0.96  Spec: 0.94  PPV: 0.87 |
| Nodule | 130 | 47.0% | 0.96 | 0.95 | 7.73% | 0.96 | 1.0 | 0.94 [0.91, 0.97] | NPV: 0.91  Sens: 0.86  Spec: 0.89  PPV: 0.81 | NPV: 0.90  Sens: 0.82  Spec: 0.94  PPV: 0.88 |
| Pneumonia | 2 | 70.1% | 1.0 | 1.0 | 0.43% | 0.03 | 1.0 | 0.97 [0.93, 1.0] | NPV: 1.0  Sens: 1.0  Spec: 0.89  PPV: 0.07 | NPV: 1.0  Sens: 1.0  Spec: 0.94  PPV: 0.13 |
| Pneumothorax | 136 | 44.8% | 0.99 | 0.99 | 7.34% | 0.96 | 1.0 | 0.98 [0.97, 0.99] | NPV: 1.0  Sens: 0.99  Spec: 0.89  PPV: 0.84 | NPV: 0.96  Sens: 0.93  Spec: 0.94  PPV: 0.90 |
| Consolidation | 83 | 52.1% | 1.0 | 1.0 | 7.62% | 0.96 | 1.0 | 0.99 [0.98, 1.0] | NPV: 1.0  Sens: 1.0  Spec: 0.89  PPV: 0.76 | NPV: 0.99  Sens: 0.96  Spec: 0.94  PPV: 0.85 |
| Edema | 23 | 64.3% | 1.0 | 1.0 | 7.84% | 0.5 | 1.0 | 0.98 [0.95, 0.99] | NPV: 1.0  Sens: 1.0  Spec: 0.89  PPV: 0.47 | NPV: 1.0  Sens: 0.96  Spec: 0.94  PPV: 0.61 |
| Emphysema | 7 | 69.0% | 0.99 | 0.86 | 0.84% | 0.5 | 1.0 | 0.94 [0.83, 1.0] | NPV: 1.0  Sens: 1.0  Spec: 0.89  PPV: 0.21 | NPV: 0.99  Sens: 0.71  Spec: 0.94  PPV: 0.26 |
| Fibrosis | 13 | 66,9% | 1.0 | 1.0 | 1.63% | 0.75 | 1.0 | 0.99 [0.97, 1.0] | NPV: 1.0  Sens: 1.0  Spec: 0.89  PPV: 0.33 | NPV: 1.0  Sens: 0.92  Spec: 0.94  PPV: 0.46 |
| Pleural Thickening | 49 | 58.4% | 1.0 | 1.0 | 3.20% | 0.89 | 1.0 | 0.99 [0.98, 1.0] | NPV: 1.0  Sens: 0.98  Spec: 0.89  PPV: 0.65 | NPV: 0.98  Sens: 0.90  Spec: 0.94  PPV: 0.76 |
| Hernia | 5 | 69.6% | 0.99 | 0.8 | 0.42% | 0.05 | 1.0 | 0.89 [0.72, 0.99] | NPV: 1.0  Sens: 0.8  Spec: 0.89  PPV: 0.13 | NPV: 1.0  Sens: 0.8  Spec: 0.94  PPV: 0.22 |
| Other | 58 | 61.0% | 0.93 | 0.78 | 1.38% | 0.75 | 1.0 | 0.78 [0.70, 0.85] | NPV: 0.87  Sens: 0.47  Spec: 0.89  PPV: 0.51 | NPV: 0.88  Sens: 0.50  Spec: 0.94  PPV: 0.67 |

**Supplementary Table 5. Comparison of DLS and radiologist performance across different subsets of (A) TB-1, (B) TB-2, (C) COV-1, (D) COV-2 with radiologist reviews from the same radiologist.**

**A**

| **TB-1** | **# cases** | **DLS High-sensitivity operating point** | | | **DLS High-specificity operating point** | | | **DLS AUC**  **[95% CI]** | **Radiologist** |
| --- | --- | --- | --- | --- | --- | --- | --- | --- | --- |
|  |  | **% predicted negative** | **NPV** | **Sensitivity** | **% predicted positive** | **PPV** | **Specificity** |  |  |
| Radiologist 1 Subset | 73 | 23 (31.51%) | 0.953 | 0.913 | 40 (54.79%) | 0.875 | 0.833 | 0.934  [0.873, 0.980] | NPV: 0.698  Sens: 0.698  Spec: 1.00  PPV: 1.00 |
| Radiologist 2 Subset | 34 | 16 (47.06%) | 0.833 | 0.813 | 12 (35.29%) | 1.0 | 1.0 | 0.906  [0.782, 0.986] | NPV: 0.762  Sens: 0.722  Spec: 1.00  PPV: 1.00 |
| Radiologist 3 Subset | 35 | 15 (42.86%) | 0.870 | 0.800 | 18 (51.43%) | 1.0 | 1.0 | 0.982  [0.932, 1.00] | NPV: 0.696  Sens: 0.632  Spec: 1.00  PPV: 1.00 |
| Radiologist 4 Subset | 85 | 33 (38.82%) | 0.978 | 0.970 | 39 (45.88%) | 0.974 | 0.975 | 0.968  [0.930, 0.994] | NPV: 0.778  Sens: 0.787  Spec: 0.925  PPV: 0.921 |
| Radiologist 5 Subset | 98 | 48 (48.98%) | 0.837 | 0.833 | 40 (40.82%) | 0.925 | 0.39 | 0.939  [0.888, 0.976] | NPV: 0.633  Sens: 0.727  Spec: 0.980  PPV: 0.969 |
| Radiologist 6 Subset | 137 | 64 (46.72%) | 0.905 | 0.906 | 53 (38.69%) | 0.962 | 0.973 | 0.947  [0.903, 0.979] | NPV: 0.683  Sens: 0.767  Spec: 0.892  PPV: 0.843 |

**B**

| **TB-2** | **# cases** | **DLS High-sensitivity operating point** | | | **DLS High-specificity operating point** | | | **DLS AUC**  **[95% CI]** | **Radiologist** |
| --- | --- | --- | --- | --- | --- | --- | --- | --- | --- |
|  |  | **% predicted negative** | **NPV** | **Sensitivity** | **% predicted positive** | **PPV** | **Specificity** |  |  |
| Radiologist 1 Subset | 36 | 19 (52.77%) | 1.00 | 1.00 | 8 (22.22%) | 0.750 | 0.931 | 0.951  [0.826, 1.00] | NPV: 0.714  Sens: 0.931  Spec: 0.931  PPV: 0.714 |
| Radiologist 2 Subset | 32 | 13 (40.63%) | 0.938 | 0.923 | 16 (50.00%) | 0.938 | 0.938 | 0.973  [0.905, 1.000] | NPV: 0.813  Sens: 0.833  Spec: 0.938  PPV: 0.929 |
| Radiologist 3 Subset | 37 | 11 (29.73%) | 1.00 | 1.00 | 16 (43.24%) | 0.875 | 0.905 | 0.967  [0.900, 1.00] | NPV: 0.813  Sens: 0.875  Spec: 1.00  PPV: 1.00 |
| Radiologist 4 Subset | 28 | 8 (28.57%) | 1.00 | 1.00 | 13 (46.43%) | 1.00 | 1.00 | 0.990  [0.952, 1.00 | NPV: 0.786  Sens: 0.824  Spec: 1.00  PPV: 1.00 |

**C**

| **COV-1** | **# cases** | **DLS High-sensitivity operating point** | | | **DLS High-specificity operating point** | | | **DLS AUC**  **[95% CI]** | **Radiologist** |
| --- | --- | --- | --- | --- | --- | --- | --- | --- | --- |
|  |  | **% predicted negative** | **NPV** | **Sensitivity** | **% predicted positive** | **PPV** | **Specificity** |  |  |
| Radiologist 1 Subset | 584 | 35 (6.00%) | 0.886 | 0.976 | 72 (12.33%) | 0.528 | 0.918 | 0.691  [0.646, 0.737] | NPV: 0.772  Sens: 0.427  Spec: 0.770  PPV: 0.425 |
| Radiologist 2 Subset | 477 | 39 (8.18%) | 0.795 | 0.944 | 67 (14.05%) | 0.507 | 0.900 | 0.687  [0.631, 0.737] | NPV: 0.799  Sens: 0.563  Spec: 0.756  PPV: 0.500 |
| Radiologist 3 Subset | 395 | 21 (5.32%) | 0.857 | 0.978 | 58 (14.68%) | 0.483 | 0.884 | 0.668  [0.613, 0.720] | NPV: 0.780  Sens: 0.603  Spec: 0.740  PPV: 0.550 |
| Radiologist 4 Subset | 363 | 14 (3.86%) | 0.929 | 0.992 | 64 (17.63%) | 0.563 | 0.878 | 0.664  [0.604, 0.722] | NPV: 0.765  Sens: 0.617  Spec: 0.722  PPV: 0.562 |

**D**

| **COV-2** | **# cases** | **DLS High-sensitivity operating point** | | | **DLS High-specificity operating point** | | | **DLS AUC**  **[95% CI]** | **Radiologist** |
| --- | --- | --- | --- | --- | --- | --- | --- | --- | --- |
|  |  | **% predicted negative** | **NPV** | **Sensitivity** | **% predicted positive** | **PPV** | **Specificity** |  |  |
| Radiologist 1 Subset | 62 | 5 (8.10%) | 0.400 | 0.897 | 12 (19.35%) | 0.667 | 0.875 | 0.625  [0.468, 0.756] | NPV: 0.576  Sens: 0.517  Spec: 0.594  PPV: 0.536 |
| Radiologist 2 Subset | 118 | 11 (9.32%%) | 0.364 | 0.885 | 12 (10.17%) | 0.833 | 0.964 | 0.656  [0.550. 0.756] | NPV: 0.574  Sens: 0.525  Spec: 0.696  PPV: 0.654 |
| Radiologist 3 Subset | 217 | 19 (8.76%) | 0.526 | 0.908 | 28 (12.90%) | 0.643 | 0.915 | 0.613  [0.534, 0.695] | NPV: 0.653  Sens: 0.571  Spec: 0.669  PPV: 0.589 |
| Radiologist 4 Subset | 208 | 24 (11.54%) | 0.708 | 0.929 | 15 (7.21%) | 0.625 | 0.725 | 0.685  [0.607, 0.751] | NPV: 0.617  Sens: 0.505  Spec: 0.725  PPV: 0.625 |

**Supplementary Table 6. Quantitative evaluation of two workflows: (A) sequential DLS-Radiologist, and (B) comparison of DLS and sequential DLS-Radiologist in distinguishing normal and abnormal CXRs across six datasets.** A, The performance of radiologist reviewing cases after DLS’ selection of abnormal CXRs for prioritized review across 6 datasets. B, Comparison of DLS and sequential DLS-Radiologist with non-inferiority test and percentages of potential caseload reduction.

**A**

| **Scenario** | **Dataset** | **Performance of Combined Radiologist + DLS (with**  **high-sensitivity operating point)** | | | | | |
| --- | --- | --- | --- | --- | --- | --- | --- |
|  |  | **No. predicted negative (%)** | **NPV** | **Sensitivity** | **No. predicted positive (%)** | **PPV** | **Specificity** |
| Abnormality detection | DS-1 | 6,622 (85.5%) | 0.85 (0.85-0.86) | 0.48 (0.46-0.50) | 1,125 (14.5%) | 0.79 (0.76-0.81) | 0.96 (0.95-0.96) |
|  |  | 6,437 (83.1%) | 0.87 (0.86-0.88) | 0.54 (0.52-0.56) | 1,310 (16.9%) | 0.76 (0.74-0.78) | 0.95 (0.94-0.95) |
|  | CXR-14 | 297 (36.7%) | 0.71 (0.66-0.76) | 0.85 (0.82-0.88) | 513 (63.3%) | 0.96 (0.94-0.98) | 0.91 (0.88-0.95) |
|  |  | 334 (41.2%) | 0.66 (0.61-0.71) | 0.80 (0.77-0.83) | 476 (58.8%) | 0.98 (0.96-0.99) | 0.95 (0.92-0.98) |
| Unseen disease: TB | TB-1 | 295 (63.9%) | 0.73 (0.68-0.78) | 0.67 (0.62-0.74) | 167 (36.1%) | 0.97 (0.94-0.99) | 0.98 (0.96-1.0) |
|  | TB-2 | 90 (67.7%) | 0.88 (0.81-0.94) | 0.79 (0.68-0.90) | 43 (32.3%) | 0.98 (0.92-1.0) | 0.99 (0.96-1.0) |
| Unseen disease: COVID-19 | COV-1 | 1,196 (65.8%) | 0.78 (0.76-0.80) | 0.55 (0.51-0.59) | 623 (34.2%) | 0.51 (0.47-0.55) | 0.75 (0.73-0.78) |
|  | COV-2 | 353 (58.3%) | 0.61 (0.57-0.66) | 0.53 (0.47-0.58) | 252 (41.7%) | 0.60 (0.55-0.66) | 0.68 (0.64-0.74) |

**B**

| **Scenario** | **Dataset** | **Number Positives** | **Radiologist**  **Sensitivity (“A”) (95% CI)** | **Radiologist + DLS**  **Sensitivity (“B”) (95% CI)** | **Delta**  **(“A”-”B”)**  **(95%CI)** | **Non-inferiority p-value** | **% caseload reduction** |
| --- | --- | --- | --- | --- | --- | --- | --- |
| Abnormality detection | DS-1 | 1845 | 0.48 (0.46-0.51) | 0.48 (0.46-0.50) | 0.005  (0.002, 0.009) | **<0.00001** | 2,313 (29.9%) |
|  |  |  | 0.54 (0.52-0.57) | 0.54 (0.52-0.56) | 0.004  (0.0001, 0.008) | **<0.00001** |  |
|  | CXR-14 | 578 | 0.87 (0.84-0.89) | 0.85 (0.82-0.88) | 0.01  (0.002, 0.02) | **<0.00001** | 194 (24.0%) |
|  |  |  | 0.81 (0.78-0.84) | 0.80 (0.77-0.83) | 0.01  (0.0008, 0.02) | **<0.00001** |  |
| Unseen disease: TB | TB-1 | 241 | 0.70 (0.65-0.76) | 0.67 (0.62-0.74) | 0.02  (0.002, 0.05) | 0.0062 | 199 (43.1%) |
|  | TB-2 | 53 | 0.79 (0.68-0.90) | 0.79 (0.68-0.90) | 0.0  (-0.05, 0.05) | **<0.00001** | 51 (38.3%) |
| Unseen disease: COVID-19 | COV-1 | 580 | 0.55 (0.51-0.59) | 0.55 (0.51-0.59) | 0.0  (-0.005, 0.005) | **<0.00001** | 109 (5.9%) |
|  | COV-2 | 288 | 0.53 (0.48-0.59) | 0.53 (0.47-0.58) | 0.003  (-0.008, 0.02) | **<0.00001** | 59 (9.8%) |

**Supplementary Table 7. Comparison between publicly available labels and majority vote labels by 3 radiologists on the CXR-14 test set (810 images).**

| Public NLP labels  Majority vote of 3 radiologists | Atelectasis | Cardiomegaly | Effusion | Infiltration | Mass | Nodule | Pneumonia | Pneumothorax | Consolidation | Edema | Emphysema | Fibrosis | Pleural Thickening | Hernia | Other* | No Finding |
| --- | --- | --- | --- | --- | --- | --- | --- | --- | --- | --- | --- | --- | --- | --- | --- | --- |
| Atelectasis | 68 | 9 | 77 | 46 | 27 | 26 | 2 | 79 | 21 | 2 | 24 | 7 | 24 | 1 | 0 | 73 |
| Cardiomegaly | 17 | 22 | 26 | 15 | 4 | 3 | 1 | 6 | 7 | 1 | 2 | 2 | 7 | 0 | 0 | 17 |
| Effusion | 42 | 5 | 85 | 33 | 24 | 20 | 3 | 47 | 18 | 1 | 10 | 5 | 23 | 1 | 0 | 55 |
| Infiltration | 8 | 1 | 13 | 13 | 8 | 6 | 1 | 16 | 6 | 1 | 6 | 1 | 2 | 0 | 0 | 11 |
| Mass | 9 | 0 | 15 | 14 | 33 | 30 | 2 | 19 | 5 | 2 | 4 | 1 | 4 | 2 | 0 | 24 |
| Nodule | 12 | 2 | 23 | 16 | 23 | 38 | 0 | 18 | 6 | 2 | 8 | 6 | 6 | 1 | 0 | 42 |
| Pneumonia | 0 | 0 | 0 | 1 | 0 | 0 | 0 | 0 | 0 | 0 | 0 | 0 | 0 | 0 | 0 | 1 |
| Pneumothorax | 19 | 0 | 21 | 10 | 15 | 13 | 1 | 76 | 3 | 1 | 23 | 1 | 5 | 0 | 0 | 29 |
| Consolidation | 15 | 2 | 28 | 16 | 11 | 7 | 2 | 10 | 12 | 0 | 2 | 0 | 6 | 0 | 0 | 24 |
| Edema | 3 | 6 | 3 | 10 | 2 | 2 | 1 | 1 | 2 | 0 | 1 | 1 | 2 | 0 | 0 | 3 |
| Emphysema | 0 | 0 | 1 | 1 | 0 | 1 | 0 | 1 | 0 | 0 | 1 | 0 | 0 | 0 | 0 | 5 |
| Fibrosis | 2 | 0 | 1 | 4 | 0 | 1 | 0 | 4 | 1 | 0 | 0 | 1 | 1 | 0 | 0 | 4 |
| Pleural Thickening | 7 | 2 | 17 | 5 | 4 | 6 | 0 | 11 | 3 | 0 | 6 | 3 | 10 | 1 | 0 | 9 |
| Hernia | 2 | 0 | 1 | 1 | 0 | 0 | 0 | 0 | 0 | 0 | 0 | 0 | 1 | 2 | 0 | 2 |
| *Other | 6 | 3 | 4 | 8 | 5 | 3 | 1 | 6 | 3 | 0 | 1 | 3 | 4 | 0 | 0 | 29 |
| No Finding | 11 | 7 | 9 | 16 | 2 | 2 | 4 | 2 | 4 | 2 | 1 | 6 | 5 | 1 | 0 | 179 |

*Note, “Other” was not part of the public labels, and one that we added to indicate findings not covered by CXR-14’s original 14 conditions, and for CXRs where the radiologists did not have a majority opinion regarding the specific finding.

**Supplementary Table 8. Radiology report pattern matching.** **(A)** Normal radiology report templates. The five-most used radiology reports to indicate that a scan is normal, along with the number of occurrences in the DS-1 train set. To obtain normal examples in our train set, we extracted all radiology reports that were used in a substantial number of cases (at least 50 occurrences) in the set and manually verified each report to ensure that it represented a “normal” scan. We then used all the images that had those radiology reports as “normal” (negative) examples in our train set. **(B)** regular expressions (natural language processing) used for identifying abnormal cases.

**A**

| Unparsed Radiology Report Template | Number of Occurrences in DS-1 Train |
| --- | --- |
| Provisional Diagnosis/Clinical Data : NILReport:: Lung fields are clear. Cardio thoracic ratio is normal. Apices, costo and cardiophrenic angles are free. Cardio vascular shadow and hila show no abnormal feature. Bony thorax shows no significant abnormality. Domes of diaphragm are well delineated.Impression  –Normal Study | 60,935 |
| Report:: Lung fields are clear. Cardio thoracic ratio is normal. Apices, costo and cardiophrenic angles are free. Cardio vascular shadow and hila show no abnormal feature. Bony thorax shows no significant abnormality. Domes of diaphragm are well delineated.Impression –Normal Study | 56,114 |
| Observation Both lung fields clear. Both hila are normal in size and position. Costophrenic and cardiophrenic angles are clear. Cardiac size and contour are within normal limits. Rib cage is normal. Soft tissues are normal.Impression Normal study. | 12,537 |
| Report:: Lung fields are clear. Cardio thoracic ratio is normal. Apices, costo and cardiophrenic angles are free. Cardio vascular shadow and hila show no abnormal feature. Bony thorax shows no significant abnormality. Domes of diaphragm are well delineated.Impression 1. Normal Study. | 9,568 |
| Report :: Lung fields are clear. Cardio thoracic ratio is normal. Apices, costo and cardiophrenic angles are free. Cardio vascular shadow and hila show no abnormal feature. Bony thorax shows no significant abnormality. Domes of diaphragm are well delineated. IMPRESSION NORMAL STUDY’ | 3,829 |

**B**

| Regular expression | Number of cases that do not contain the regular expression in DS-1 train |
| --- | --- |
| “Normal\s+study” | 39,628 |
| “Impression.+no significant abnormality” | 45,387 |

**Supplementary Table 9. Performance comparison of various modeling decisions on the DS-1 tune split: (A) different network Architectures, (B) image resolutions, and (C) pretraining on ImageNet vs training from random initialization.**

**A**

|  | MobileNet | Xception | EfficientNet-B5 | EfficientNet-B8 | EfficientNet-B7 |
| --- | --- | --- | --- | --- | --- |
| AUC (95%CI) | 0.891  (0.885, 0.898) | 0.912  (0.909, 9.16) | 0.932  (0.928, 0.937) | 0.941  (0.934, 0.947) | 0.972  (0.967, 0.976) |

**B**

| Resolution | 128x128 | 256x256 | 512x512 | 600x600 | 1024x1024 |
| --- | --- | --- | --- | --- | --- |
| AUC (95%CI) | 0.573 (0.560-0.585) | 0.880 (0.872-0.888) | 0.965 (0.959-0.970) | 0.972  (0.967, 0.976) | 0.957 (0.952-0.963) |

**C**

|  | ImageNet pretrained | Trained from random initialization |
| --- | --- | --- |
| AUC (95%CI) | 0.972 (0.967, 0.976) | 0.950 (0.944, 0.955) |
